# Decreased memory B cell frequencies in COVID‐19 delta variant vaccine breakthrough infection

**DOI:** 10.15252/emmm.202115227

**Published:** 2022-01-21

**Authors:** Matthew Zirui Tay, Angeline Rouers, Siew‐Wai Fong, Yun Shan Goh, Yi‐Hao Chan, Zi Wei Chang, Weili Xu, Chee Wah Tan, Wan Ni Chia, Anthony Torres‐Ruesta, Siti Naqiah Amrun, Yuling Huang, Pei Xiang Hor, Chiew Yee Loh, Nicholas Kim‐Wah Yeo, Bei Wang, Eve Zi Xian Ngoh, Siti Nazihah Mohd Salleh, Jean‐Marc Chavatte, Alicia Jieling Lim, Sebastian Maurer‐Stroh, Lin‐Fa Wang, Raymond Valentine Tzer Pin Lin, Cheng‐I Wang, Seow‐Yen Tan, Barnaby Edward Young, Yee‐Sin Leo, David C Lye, Laurent Renia, Lisa FP Ng

**Affiliations:** ^1^ A*STAR Infectious Diseases Labs Agency for Science, Technology and Research (A*STAR) Singapore City Singapore; ^2^ Singapore Immunology Network A*STAR Singapore City Singapore; ^3^ Programme in Emerging Infectious Diseases Duke‐NUS Medical School Singapore City Singapore; ^4^ National Centre for Infectious Diseases Singapore City Singapore; ^5^ National Public Health Laboratory National Centre for Infectious Diseases Singapore City Singapore; ^6^ Bioinformatics Institute A*STAR Singapore City Singapore; ^7^ SingHealth Duke‐NUS Global Health Institute Singapore City Singapore; ^8^ Department of Microbiology and Immunology Yong Loo Lin School of Medicine National University of Singapore Singapore City Singapore; ^9^ Department of Infectious Diseases Changi General Hospital Singapore City Singapore; ^10^ Department of Infectious Diseases Tan Tock Seng Hospital Singapore City Singapore; ^11^ Lee Kong Chian School of Medicine Nanyang Technological University Singapore City Singapore; ^12^ Yong Loo Lin School of Medicine National University of Singapore and National University Health System Singapore City Singapore; ^13^ Department of Biochemistry Yong Loo Lin School of Medicine National University of Singapore Singapore City Singapore; ^14^ National Institute of Health Research Health Protection Research Unit in Emerging and Zoonotic Infections University of Liverpool Liverpool UK; ^15^ Institute of Infection, Veterinary and Ecological Sciences University of Liverpool Liverpool UK

**Keywords:** correlate of risk, COVID‐19, delta, memory B cells, vaccine breakthrough, Immunology, Microbiology, Virology & Host Pathogen Interaction

## Abstract

The SARS‐CoV‐2 Delta (B.1.617.2) variant is capable of infecting vaccinated persons. An open question remains as to whether deficiencies in specific vaccine‐elicited immune responses result in susceptibility to vaccine breakthrough infection. We investigated 55 vaccine breakthrough infection cases (mostly Delta) in Singapore, comparing them against 86 vaccinated close contacts who did not contract infection. Vaccine breakthrough cases showed lower memory B cell frequencies against SARS‐CoV‐2 receptor‐binding domain (RBD). Compared to plasma antibodies, antibodies secreted by memory B cells retained a higher fraction of neutralizing properties against the Delta variant. Inflammatory cytokines including IL‐1β and TNF were lower in vaccine breakthrough infections than primary infection of similar disease severity, underscoring the usefulness of vaccination in preventing inflammation. This report highlights the importance of memory B cells against vaccine breakthrough and suggests that lower memory B cell levels may be a correlate of risk for Delta vaccine breakthrough infection.

## Graphical abstract

This study compares the immune characteristics of 55 patients with vaccine breakthrough SARS‐CoV‐2 infection and 86 uninfected vaccinated close contacts. Memory B cell levels differed between the groups, identifying them as potential immune correlates of protection in Delta vaccine breakthrough.

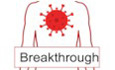

**The paper explained**

**Problem**

Vaccination has been a key tool for disease reduction in the current COVID‐19 pandemic. However, the SARS‐CoV‐2 Delta variant can cause infection in vaccinated persons, resulting in continued viral transmission even in populations with high vaccination rates. It is unclear whether some vaccinated persons are more susceptible to Delta variant infection compared to others, and if so, what factors explain the difference in susceptibility.

**Results**

We hypothesized that levels of particular immune markers or cells would be lower in infected individuals, which may have contributed toward their susceptibility to infection relative to the uninfected close contacts. We identified a cohort of patients who were infected despite being vaccinated (vaccine breakthrough infections) and compared their immunological characteristics to their vaccinated close contacts who were not infected. We found that while levels of antibodies were similar in both groups, memory B cell levels were lower in the vaccine breakthrough cases. We further investigated the quality of antibodies derived from memory B cells and found that they retained more neutralization effectiveness against the Delta variant as compared with circulating antibody in the plasma.

**Impact**

These results highlight the potential role of memory B cells in protection from Delta vaccine breakthrough infection. The results suggest that memory B cell levels may be a correlate of protection against Delta variant infection in vaccinated populations—if so, this will be useful for determining the level of susceptibility in a population. It will also be useful in the design of future vaccines or vaccine boosters.

## Introduction

The ongoing severe acute respiratory syndrome coronavirus 2 (SARS‐CoV‐2) pandemic has caused more than 300,000 confirmed infections worldwide daily in the first half of 2021. Efficacy of licensed SARS‐CoV‐2 vaccines range from 50 to 95% depending on vaccine type and infection variant (Kim *et al*, 2021; McDonald *et al*, 2021). Thus, while vaccination markedly decreases the chances of infection and severe disease, breakthrough symptomatic and asymptomatic infections do occur. The duration of protective immunity after vaccination is uncertain, and it is unclear whether breakthrough infections are due to immune evasion by Variants of Concern (VOCs) or vaccine failure to elicit a protective immune response in some individuals (Abu‐Raddad *et al*, 2021; Sheikh *et al*, 2021). In order to predict how frequently vaccine breakthrough infections could occur, and how public health outcomes would change depending on waning vaccine‐mediated immunity levels, there is an urgent need to understand the immune parameters that are correlates of risk for SARS‐CoV‐2 vaccine breakthrough.

Recent analyses of neutralizing and binding antibody responses from cohort studies (Bergwerk *et al*, 2021; Feng *et al*, 2021; preprint: Gilbert *et al*, 2021) and aggregated clinical trial data (Earle *et al*, 2021; Khoury *et al*, 2021) have found correlations between antibody levels and protection from symptomatic infection. However, other potentially relevant immune parameters have not yet been investigated. Multi‐parameter investigations into the levels of other important immune factors, including memory B cells and T cells, are necessary in order to better define the immune parameters that correlate with risk of vaccine breakthrough infection. In addition, correlates of risk may be different in the context of the Delta (B.1.617.2) variant which has now become the dominant strain globally (Campbell *et al*, 2021; World Health Organization, 2021), and shows escape from vaccine‐elicited neutralizing antibody responses (Planas *et al*, 2021). Such correlates of risk may also be mediators of protection (Plotkin, 2010), which can be further investigated to better understand the mechanisms of vaccine‐induced protection, informative for future vaccine development.

Due to extensive contact tracing efforts in Singapore, there was an opportunity to identify fully vaccinated patients who developed vaccine breakthrough infections and displayed mild symptoms or were asymptomatic. The aim of this study was to characterize the immune parameters present in this cohort of patients with mostly Delta (B.1.617.2) vaccine breakthrough infections, comparing them against those of vaccinated but uninfected close contacts in order to discern potential differences that may be correlates of risk for vaccine breakthrough.

## Results

Plasma and peripheral blood mononuclear cell (PBMC) samples were collected from 55 vaccine breakthrough cases (defined as individuals who became PCR positive at least 2 weeks after two vaccine doses) and 86 of their vaccinated and uninfected close contacts in Singapore that occurred between April and June 2021 (Table 1). Sample collection was done as soon as possible after diagnosis (maximum 7 days, and median 3 days from onset of symptoms to sample collection) (Table 1). The majority of cases (87.3%, 48/55) were identified as Delta (B.1.617.2) variant vaccine breakthrough infection via epidemiological data and direct sequencing. All participants received the Pfizer‐BioNTech BNT162b2 vaccine.

**Table 1:** Demographics of participants.

	Vaccine breakthrough	Close contacts	Primary infection
	*n* = 55	*n* = 86	*n* = 49
Female sex	34.5% (19)	87.2% (75)	32.6% (16)
Age, median (IQR)	46 (36.5–59.5)	31 (26.25–35.75)	32 (26–45)
Any comorbidity^a^	10.9% (6)	9.3% (8)	37.0% (17)
Days from symptom onset or exposure to sample collection, median (IQR)^b^	3 (2–4.5)	9 (7–12)	3 (2–4)
Days from second dose of BNT162b2 to symptom onset or exposure, median (IQR)^c^	82 (51.5–99)	68 (64–70)	NA
Ethnicity			
Chinese	45.5% (25)	45.3% (39)	38.8% (19)
Malay	9.1% (5)	8.1% (7)	14.3% (7)
Indian	29.1% (16)	14.0% (12)	20.4% (10)
Others	16.4% (9)	32.6% (28)	34.7% (17)
Breakthrough variant			
Delta	87.3% (48)	NA	0% (0)
Non‐Delta	5.5% (3)	NA	100% (49)
Unknown	7.3% (4)	NA	0% (0)
Severity of disease			
Asymptomatic	21.8% (12)	NA	14.3% (7)
Mild symptoms	78.2% (32)	NA	85.7% (42)
Severe symptoms	0% (0)	NA	0% (0)

^a^ Participants were asked if they had any of the following specific comorbidities; myocardial infarction, congestive heart failure, asthma, other chronic lung diseases, rheumatologic disease, chronic liver disease, diabetes, chronic renal disease, malignancies, or AIDS/HIV.

^b^ Only samples collected at most 7 days post‐symptom onset were included in the cohort. If patients were asymptomatic, date of first PCR positive was used instead.

^c^ Only patients > 14 days past second vaccination were included in the cohort. If patients were asymptomatic, date of first PCR positive was used instead.

### Plasma antibody responses do not differ between vaccine breakthrough cases and vaccinated close contacts

**Figure EV1:**
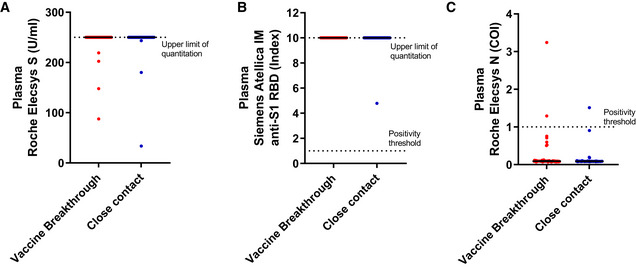
Anti‐SARS‐CoV‐2 titers in commercial serological assays Anti‐SARS‐CoV‐2 S protein antibodies in plasma of vaccine breakthrough cases (*n* = 55) or close contacts (*n* = 86) were determined by Roche Elecsys® S antibody assay. Values above the upper limit of quantitation, 250 U/ml, are truncated to 250 U/ml.Anti‐SARS‐CoV‐2 S1 RBD antibodies in plasma of vaccine breakthrough cases (*n* = 55) or close contacts (*n* = 86) determined by Siemens Atellica® IM SARS‐CoV‐2 Total (COVT) assay. Values above the upper limit of quantitation, 10, are truncated to 10.Anti‐SARS‐CoV‐2 N protein antibodies in plasma of vaccine breakthrough cases (*n* = 55) or close contacts (*n* = 86) determined by Roche Elecsys® N antibody assay. Data information: Dotted lines indicate upper limit of quantitation (A,B) or positivity threshold (B,C) based on manufacturer’s instructions. Error bars indicate median and interquartile range. Source data are available online for this figure.

We first investigated the levels of plasma antibodies against the Spike (S) protein. 100% (55/55) of vaccine breakthrough cases and 100% (86/86) of their close contacts were positive for both the Roche Elecsys^®^ Anti‐SARS‐CoV‐2 S and Siemens Atellica^®^ IM SARS‐CoV‐2 Total (COV2T) commercial serological assays (Fig EV1A and B). These data suggest that pre‐existing antibody responses were present and that these were true vaccine breakthrough cases and not cases of vaccine failure. Conversely, only 3.6% (2/55) of vaccine breakthrough cases and 1.2% (1/86) of close contacts were positive for the Roche Elecsys^®^ Anti‐SARS‐CoV‐2 nucleocapsid (N) commercial serological assay (Fig EV1C).

**Figure 1:**
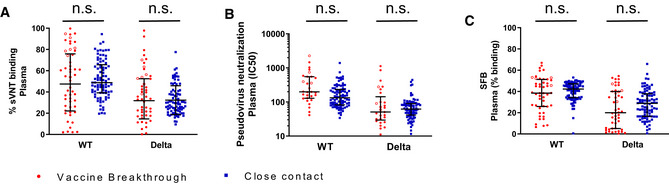
Similar plasma antibody levels against SARS‐Cov‐2 between vaccine breakthrough cases and close contacts Anti‐SARS‐CoV‐2 neutralizing antibodies in plasma of vaccine breakthrough cases (*n* = 54) and close contacts (*n* = 86) were measured by surrogate virus neutralization test (sVNT). The percentage inhibition of ACE2 binding to RBD at plasma dilution of 1:800 is given. The wild‐type (Wuhan) strain or Delta (B.1.617.2) strain RBD was used, respectively.Anti‐SARS‐CoV‐2 neutralizing antibodies in plasma of vaccine breakthrough cases (*n* = 29) and close contacts (*n* = 86) were measured by pseudovirus neutralization assay.Anti‐SARS‐CoV‐2 S protein antibodies in plasma of vaccine breakthrough cases (*n* = 48) and close contacts (*n* = 86) were determined by S protein flow cytometry‐based assay (SFB). The percentage of plasma antibody‐bound cells out of the total population of cells transfected with membrane‐anchored SARS‐CoV‐2 S protein is given. In all graphs, error bars denote median and interquartile range. Red solid circles indicate Delta strain vaccine breakthrough, and red open circles indicate non‐Delta or unknown strain. Data information: Statistical comparisons were determined by one‐tailed Mann–Whitney *U*‐test. Error bars indicate median and interquartile range. Source data are available online for this figure.

Several Variants of Concern (VOCs) have emerged in the course of the COVID‐19 pandemic, including Alpha (B.1.1.7), Beta (B.1.351), Gamma (P.1), and Delta (B.1.617.2). Several studies have shown reduced vaccine efficacy in populations where the predominant infection strain is a VOC (Abu‐Raddad *et al*, 2021; Kustin *et al*, 2021; Lopez Bernal *et al*, 2021; preprint: Nasreen *et al*, 2021; Skowronski *et al*, 2022). A prior study has suggested that in the context of Alpha strain infection, lower neutralizing antibody titers correlated with the occurrence of breakthrough infections (Bergwerk *et al*, 2021). To determine whether neutralizing antibody titers also correlated with vaccine breakthrough risk in our cohort, we examined neutralizing antibody levels against both wild‐type (WT) and Delta strains. We measured neutralizing plasma antibody responses via the surrogate virus neutralization test (sVNT), which measures inhibition of the binding between recombinant RBD protein and angiotensin‐converting enzyme 2 (ACE2) (Wacharapluesadee *et al*, 2021). Interestingly, vaccine breakthrough cases did not display lower levels of neutralizing antibodies compared with close contacts for both the WT and Delta strains (Fig 1A). To further verify this, neutralizing plasma antibody responses were quantified via the SARS‐CoV‐2 pseudovirus neutralization assay and also the total IgG binding responses against membrane‐anchored S‐antigen via the S protein flow cytometry‐based assay (SFB) (Goh *et al*, 2021a, 2021b). In both assays, plasma antibodies in vaccine breakthrough cases were not higher than close contacts (Fig 1B and C). Together, these data suggest that the vaccine breakthrough cases in our cohort did not have inferior plasma antibody responses against either the vaccine or breakthrough strains.

### Vaccine breakthrough cases have lower frequencies of RBD‐specific memory B cells than vaccinated close contacts

**Figure 2:**
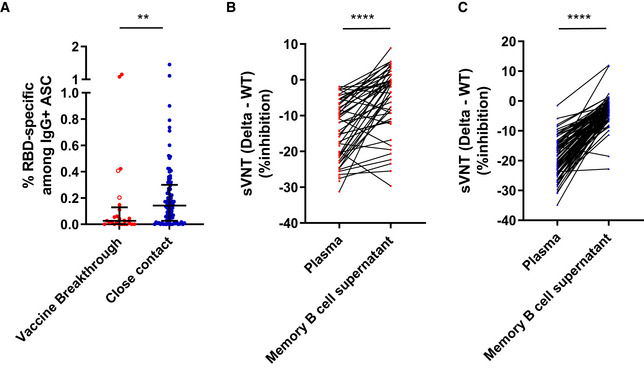
Memory B cell responses against SARS‐CoV‐2 in vaccine breakthrough cases and close contacts AFrequencies of memory B cells specific for SARS‐CoV‐2 RBD are examined via ELISpot for vaccine breakthrough cases (*n* = 25) and close contacts (*n* = 86). The frequency of RBD‐specific memory B cells is given as a percentage of the total IgG‐secreting B cell population. *P* value was determined by two‐tailed Mann–Whitney *U*‐test, **P < 0.01. Error bars indicate median and interquartile range.B, CLevels of anti‐SARS‐CoV‐2 neutralizing antibodies inhibiting ACE2 binding to WT or Delta are determined by surrogate virus neutralization test (sVNT), in supernatant from 5‐day culture of activated memory B cells and in plasma. The difference in sVNT % inhibition between WT and Delta is taken, and this difference is compared between plasma and memory B cell supernatant for vaccine breakthrough cases (*n* = 48 pairs) (B) and for close contacts (*n* = 86 pairs) (C). Data information: *P* values for paired comparisons were determined by two‐tailed Wilcoxon matched‐pairs signed rank test, ****P < 0.0001. In all graphs, error bars denote median and interquartile range. Red solid circles indicate Delta strain vaccine breakthrough, and red open circles indicate non‐Delta or unknown strain. Source data are available online for this figure.

In addition to circulating plasma antibody, memory B cells can play an important role in long‐lasting immunity, including against heterologous influenza infection (Leach *et al*, 2019) and bacterial pneumonia (Barker *et al*, 2021). Thus, the frequencies of circulating SARS‐CoV‐2‐specific memory B cells were investigated via memory B cell ELISpot targeted to the SARS‐CoV‐2 receptor‐binding domain (RBD). In contrast to plasma antibody levels, the frequency of RBD‐specific memory B cells was lower in vaccine breakthrough cases than in uninfected close contacts (Fig 2A). This was unlikely to be due to demographic differences between cohorts, since the frequencies of RBD‐specific memory B cells did not correlate with age, gender, or ethnicity in either the vaccine breakthrough cases or their close contacts (Appendix Fig S1). Thus, lower circulating memory B cell levels may be a correlate of risk for vaccine breakthrough.

We further characterized the potential neutralization efficiency of antibodies secreted by memory B cells, by analyzing the levels of secreted antibody after 5 days of *ex vivo* stimulation with IL‐2 and R848 (TLR7/8 ligand). Memory B cell supernatants containing secreted antibody were analyzed by multiplex sVNT to examine RBD‐targeted neutralizing responses. Interestingly, compared to their close contacts, vaccine breakthrough cases showed higher levels of memory B cell‐secreted neutralizing antibodies against both WT and Delta strains (Appendix Fig S2A). Similar observations were also found for S‐specific binding antibodies measured by both the Roche S and SFB assays (Appendix Fig S2B and C). This increase may be due to memory B cell priming by ongoing infection in the vaccine breakthrough cases, which would indicate that vaccine‐elicited memory B cells can be efficiently reactivated by Delta variant infection.

Memory B cells typically represent a more diverse antigen‐specific population relative to plasmablasts and long‐lived plasma cells (Akkaya *et al*, 2020). Thus, the degree of cross‐reactivity in memory B cell responses relative to plasma responses was assessed. To measure the level of neutralization cross‐reactivity across variants, the relative reduction in neutralization efficiency was calculated by subtracting the sVNT response against WT from the sVNT response against Delta. Compared to plasma, memory B cell‐secreted antibodies retained more efficient neutralization against Delta (Fig 2B and C). This was observed in both vaccine breakthrough and vaccinated close contacts, suggesting that it is a feature of memory B cells derived from BNT162b2 vaccination. A similar trend was seen when comparing SFB plasma and memory B cell responses against each strain (Appendix Fig S2D). Together, these data suggest that antibodies from activated memory B cells are more frequently capable of cross‐neutralization against Delta, as compared with plasma antibody.

**Figure EV2:**
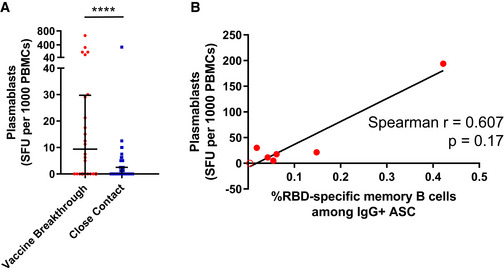
SARS‐CoV‐2 RBD‐specific plasmablasts are increased in vaccine breakthrough participants Frequencies of plasmablasts specific for SARS‐CoV‐2 RBD are examined via ELISpot for vaccine breakthrough cases (*n* = 24) and close contacts (*n* = 86). The frequency of RBD‐specific memory B cells is given as the number of plasmablasts (as determined by spot‐forming units, SFU) per 1,000 PBMCs plated. Error bars denote median and interquartile range. *P* value for unpaired comparison was determined by two‐tailed Mann–Whitney *U*‐test, ****P < 0.0001.The relationship between SARS‐CoV‐2 RBD‐specific plasmablast and memory B cell frequencies in vaccine breakthrough cases was quantified via ELISpot is shown, and correlation was determined by Spearman correlation (*n* = 7 pairs). Red solid circles indicate Delta strain vaccine breakthrough, and red open circles indicate non‐Delta or unknown strain. Source data are available online for this figure.

To better understand the reactivation of B cell responses during breakthrough infection, we also investigated antibody‐secreting plasmablast responses in a subset of the vaccine breakthrough cases and close contacts. SARS‐CoV‐2 RBD‐specific plasmablasts were significantly higher in vaccine breakthrough cases than close contacts, as measured by antigen‐specific IgG ELISpot (Fig EV2A). However, inverse correlation between memory B cells and plasmablast levels was not observed, suggesting that the reduced levels of memory B cells in vaccine breakthrough cases were not because they had differentiated into plasmablasts during ongoing infection (Fig EV2B).

### Differences in CD4^+^ and CD8^+^ T cell subsets between vaccine breakthrough cases compared to vaccinated close contacts

**Figure 3:**
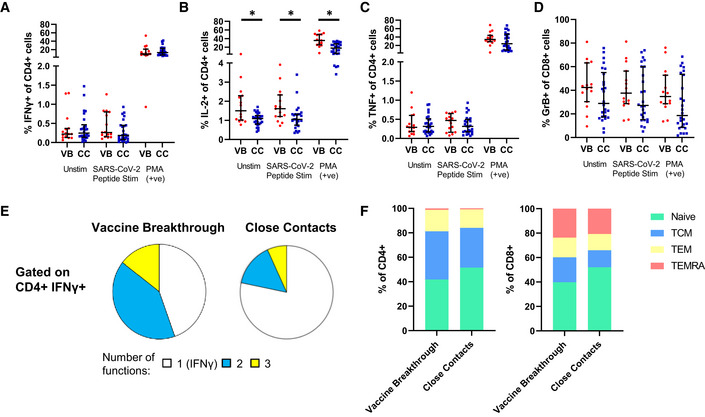
T cell responses in vaccine breakthrough cases and close contacts A–DPBMCs from vaccine breakthrough cases (*n* = 15) and close contacts (*n* = 26) were examined for T cell responses. PBMCs were left unstimulated (Unstim), were stimulated with pooled SARS‐CoV‐2 PepTivator^®^ S, S1, M, and N peptides for 6 h (SARS‐CoV‐2 Peptide Stim), or non‐specifically stimulated with phorbol 12‐myristate 13‐acetate (PMA), then assessed by high‐dimensional flow cytometry. CD4^+^ T cells positive for intracellular staining of IFNγ (a), IL‐2 (b), TNF (c), or CD8^+^ T cells positive for intracellular staining of granzyme B (d) are shown. Error bars indicate median and interquartile range.ETo examine polyfunctionality of CD4^+^ T cells, SARS‐CoV‐2 peptide‐stimulated IFNγ^+^ CD4^+^ T cells were further examined for co‐expression of IL‐2 and/or TNF, and the fraction of cells expressing IFNγ only (1 function), IFNγ and either IL‐2 or TNF (2 functions), or IFNγ and both IL‐2 and TNF (3 functions) are shown.FThe differentiation status of SARS‐CoV‐2 peptide‐stimulated CD4^+^ T cells (left) and CD8^+^ T cells (right) were compared based on CD27 and CD45RA expression (Naïve: CD27^+^ CD45RA^+^; T central memory (TCM): CD27^+^ CD45RA^−^; T effector memory (TEM): CD27^−^ CD45RA^−^; TEMRA: CD27^−^ CD45RA^+^). The average of all vaccine breakthrough cases or close contacts is plotted, respectively. Data information: *P* value for unpaired comparison was determined by two‐tailed Mann–Whitney *U*‐test, **P < 0.01. Source data are available online for this figure.

**Figure EV3:**
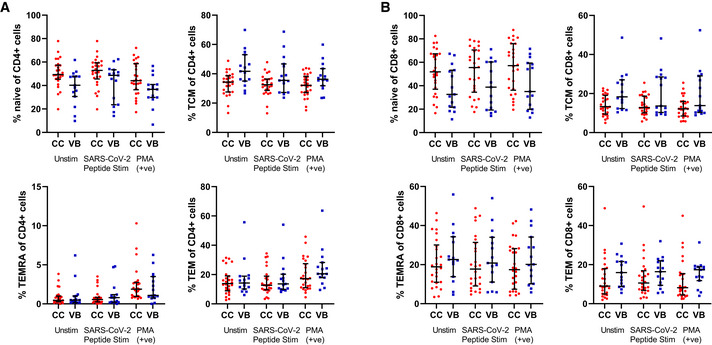
Differentiation status of T cells by individual A, BPBMCs from vaccine breakthrough cases (*n* = 15) and close contacts (*n* = 26) were examined for T cell responses. PBMCs were left unstimulated (Unstim), were stimulated with pooled SARS‐CoV‐2 PepTivator^®^ S, S1, M, and N peptides for 6 h (SARS‐CoV‐2 Peptide Stim), or non‐specifically stimulated with phorbol 12‐myristate 13‐acetate (PMA), then assessed by high‐dimensional flow cytometry. The differentiation status of CD4^+^ T cells (A) and CD8^+^ T cells (B) in unstimulated, SARS‐CoV‐2 peptide‐stimulated, and PMA‐stimulated conditions were compared based on CD27 and CD45RA expression (Naïve: CD27^+^ CD45RA^+^; T central memory (TCM): CD27^+^ CD45RA^−^; T effector memory (TEM): CD27^−^ CD45RA^−^; TEMRA: CD27^−^ CD45RA^+^). Error bars indicate median and interquartile range. Source data are available online for this figure.

T cell‐mediated immunity is another important arm of antiviral defense (Chen & Kolls, 2013). We sought to determine whether CD4^+^ and CD8^+^ T cell profiles were distinct between vaccine breakthrough cases and their close contacts. To investigate the frequencies of virus‐specific effector and memory T cells, PBMCs were stimulated *in vitro* using a library of SARS‐CoV‐2‐derived peptide antigens, followed by immune phenotyping using a high‐dimensional flow cytometry panel. Intracellular staining for cytokines and effector molecules (CD4^+^: IFNγ, IL‐2, TNF; CD8^+^: granzyme B) was performed (Appendix Table S1, Appendix Fig S3) (Seder *et al*, 2008). Results showed that CD4^+^ T cells (CD45^+^ CD4^+^ Vδ1^−^ Vδ2^−^) of vaccine breakthrough cases and close contacts were similar in their expression of IFNγ, and TNF, but vaccine breakthrough cases showed higher frequencies of CD4^+^ IL‐2^+^ cells than close contacts (Fig 3A–C). Among CD8^+^ T cells, frequencies of granzyme B^+^ cells were similar between vaccine breakthrough cases and close contacts (Fig 3D). Since a polyfunctional response has been associated with effective T cell responses, we examined the frequencies of CD4^+^ IFNγ^+^ T cells that were also IL‐2^+^ and/or TNF^+^ (Fig 3E). Vaccine breakthrough cases had higher frequencies of double‐ and triple‐positive CD4^+^ T cells than close contacts, largely driven by the increased frequency of CD4^+^ IL2^+^ T cells in vaccine breakthrough cases. We next examined the differentiation status of peptide‐stimulated T cells based on CD27 and CD45RA expression (Figs 3F and EV3). Vaccine breakthrough cases and close contacts showed similar frequencies of naïve, T central memory, T effector memory, and TEMRA cells in both CD4^+^ and CD8^+^ compartments, though there was a trend toward higher T central memory cells in vaccine breakthrough cases. Thus, vaccine breakthrough cases were broadly similar in T cell profile to close contacts, with only subtle differences observed.

### Cytokine responses in vaccine breakthrough cases demonstrate non‐inflammatory profile

**Figure 4:**
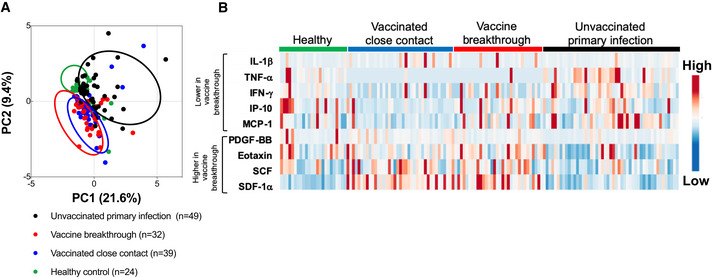
Plasma immune mediator levels of COVID‐19 vaccine breakthrough cases Levels of specific immune mediators in the first plasma samples collected during hospitalization or quarantine were quantified using a 45‐plex microbead‐based immunoassay. PCA of 45 immune mediator levels analyzed in COVID‐19 vaccine breakthrough cases (*n* = 39) compared to their vaccinated close contacts (*n* = 32), unvaccinated COVID‐19 patients with primary infection (*n* = 49), and healthy unvaccinated controls (*n* = 24). PC1 explains 21.6% of the variation, while PC2 explains 9.4% of the variation; color denotes different groups of subjects.Heat map of selected immune mediator levels in plasma samples of healthy controls, vaccinated close contacts, COVID‐19 vaccine breakthrough cases, and unvaccinated COVID‐19 patients with primary infection. Each color represents the relative concentration of a particular analyte. Blue and red indicate low and high concentration, respectively. Source data are available online for this figure.

**Figure EV4:**
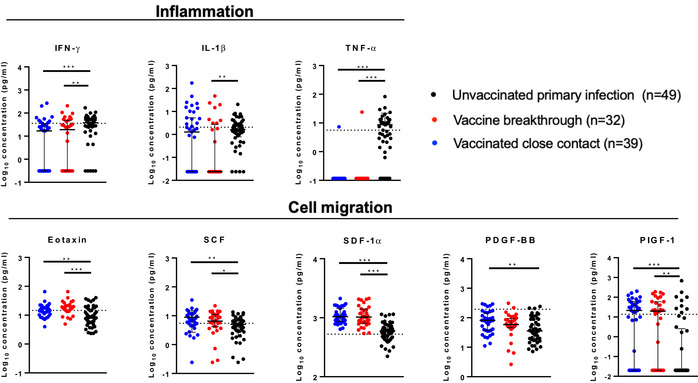
Cytokine responses in vaccine breakthrough cases and close contacts show milder inflammatory profile compared to primary infection in unvaccinated persons Cytokine levels in vaccine breakthrough cases, close contacts, and a matched primary infection cohort were determined by Luminex assay. Selected inflammation‐related (top row) and cell migration‐related (bottom row) cytokines are shown. Dotted lines represent the average response in a population of healthy controls. In all graphs, error bars denote median and interquartile range. *P* values for unpaired comparisons were determined by two‐tailed Mann–Whitney *U*‐test, *P < 0.05, **P < 0.01, ***P < 0.001. Source data are available online for this figure.

**Figure EV5:**
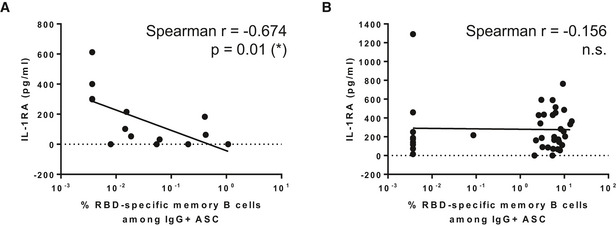
Correlation between IL‐1RA levels and SARS‐CoV‐2 RBD‐specific memory B cells A, BThe relationship between IL‐1RA and log‐transformed SARS‐CoV‐2 RBD‐specific memory B cell responses is shown for vaccine breakthrough patients (A) and in their close contacts (B). Dotted lines represent the baseline of 0. Correlation was determined by Spearman correlation, *P < 0.05. Source data are available online for this figure.

To determine whether there were differences in inflammatory markers in vaccine breakthrough infection relative to primary infection, the levels of 45 different cytokines were also measured in the plasma of vaccine breakthrough cases and their close contacts, compared to unvaccinated patients with primary infection of matched disease severity from our earlier cohort (Table 1) (Young *et al*, 2020). Vaccine breakthrough cases had systemic cytokine profiles similar to their uninfected close contacts, but distinct from the primary infection (Fig 4A). Notably, cytokines commonly associated with more severe disease, including IL‐1β, TNF, and IFNγ, were significantly lower compared with a previous cohort of unvaccinated patients with primary infection with matched disease symptoms (either mild symptoms or no symptoms) (Table 1; Figs 4B and EV4). Chemokines including Eotaxin, SCF, SDF‐1α, and PIGF‐1, associated with immune cell migration, were also significantly lower in vaccine breakthrough cases than in patients with primary infection (Figs 4B and EV4). Interestingly, healthy unvaccinated controls clustered away from both primary infection patients and vaccine breakthrough/vaccinated close contacts, suggesting different effects of infection and vaccination on innate immunity. Levels of IL‐1RA were negatively correlated with memory B cell responses in vaccine breakthrough cases (Spearman correlation *r* = −0.674, *P* = 0.01) but not in close contacts (*r* = −0.156) (Fig EV5).

## Discussion

Here, we examine vaccine breakthrough in‐depth, characterizing multiple humoral and cellular immune parameters in parallel. Differences in virus‐specific plasma antibody levels between vaccine breakthrough cases and their close contacts were not observed, despite using several independent assays to examine neutralization and binding to spike protein or RBD. These are in agreement with other small studies of vaccine breakthrough cases, which found no differences or marginal differences at the plasma antibody level (Hacisuleyman *et al*, 2021; Mlcochova *et al*, 2021; Rovida *et al*, 2021). However, this is in contrast to recent studies from Israel (Bergwerk *et al*, 2021) and the UK (Feng *et al*, 2021) showing lower neutralizing antibody titers in patients with breakthrough Alpha variant infection and also meta‐analyses showing a strong correlation between plasma antibody levels and vaccine efficacy against symptomatic infection across different vaccine types and populations (Earle *et al*, 2021; Khoury *et al*, 2021). Several reasons for this are plausible. First, the majority of cases in our study had Delta variant infection. The Delta variant is known for a greater degree of escape from vaccine‐elicited neutralizing antibody responses (Planas *et al*, 2021), thus vaccine‐elicited plasma antibodies may be less efficient for protection with different correlates of risk. Second, our study included several asymptomatic cases. Notably, in the above‐mentioned vaccine efficacy study from the UK (Feng *et al*, 2021), no correlation was found between plasma antibody responses and asymptomatic infection, unlike with symptomatic infection (Feng *et al*, 2021)—different immune mechanisms may be involved to prevent asymptomatic infection.

The lower frequency of virus‐specific memory B cells identified in vaccine breakthrough patients suggests that memory B cell levels against SARS‐CoV‐2 could be a correlate of risk for vaccine breakthrough. Previous studies have suggested that the additional diversity found in memory B cells provides a secondary layer of defense against variant pathogens that are able to escape plasma antibodies (Purtha *et al*, 2011; Leach *et al*, 2019; Akkaya *et al*, 2020). In line with this, we found that antibodies from activated memory B cells retained a higher fraction of their neutralizing and binding efficiency against the Delta variant, as compared to plasma antibodies. This is concordant with other recent results showing that 30‐50% of monoclonal antibodies derived from memory B cells effectively bind or neutralize VOCs after BNT162b2 vaccination (Sokal *et al*, 2021). This suggests a potential mechanism linking memory B cells to protection against VOCs that are neutralized less efficiently by the plasma antibody response. Low frequency and/or diversity of the specific memory B cell population during exposure to SARS‐CoV‐2 may lead to vaccine breakthrough.

BNT162b2 vaccination elicits T cell responses (Guerrera *et al*, 2021), and such T cells may contribute to protection. Notably, early induction of functional anti‐SARS‐CoV‐2 T cells after infection has been associated with rapid viral clearance and mild disease (Tan *et al*, 2021), raising the hypothesis that a robust T cell response could prevent virus establishment and infection. In our study, T cell responses appeared broadly similar across vaccine breakthrough and close contacts. Even though polyfunctional T cells are associated with better disease control in various infection settings (Seder *et al*, 2008), we did not identify a lack of polyfunctionality in the CD4^+^ T cells of vaccine breakthrough cases.

Vaccine breakthrough cases showed cytokine profiles that were remarkably similar to the uninfected vaccinated close contact cohort and dissimilar to patients who contracted primary infection with mild symptoms. This provides further evidence for immune‐mediated protection against virus‐induced inflammation and disease despite vaccine breakthrough infection. Notably, memory B cell responses were negatively correlated with IL‐1RA levels in vaccine breakthrough cases but not in close contacts. IL‐1RA has been previously associated with disease severity (Zhao *et al*, 2020), suggesting that memory B cell responses may play a role in alleviating inflammation and disease severity despite vaccine breakthrough.

The primary limitation of the current study is in its retrospective design and lack of pre‐infection immune status. Such studies will not be able to rule out confounding effects due to early events in the response to infection. These include boosts to antibody levels due to activation of antibody‐secreting cells, as well as immediate changes to circulating memory B cell levels and T cell levels due to recruitment into germinal centers or other lymphoid organs. In particular, reduced memory B cell levels in vaccine breakthrough cases may have been due to recruitment of memory B cells out of circulation after activation by infection. The current study provides additional impetus for future prospective studies to closely examine memory B cell and T cell responses. Studies have indicated that memory B cell responses are longer lasting compared with plasma antibody levels following natural infection (Dan *et al*, 2021; Sakharkar *et al*, 2021; Turner *et al*, 2021). Memory B cell responses are also effectively elicited by vaccination (Goel *et al*, 2021; Piano Mortari *et al*, 2021), though their longevity relative to plasma antibody remains an open question. Protective effects of memory B cells may become more apparent with increased time since vaccination, as plasma antibody levels wane. Should vaccine‐elicited memory B cell responses prove to be long‐lived, and also a mechanistic correlate of protection, vaccines may provide a longer duration of protection compared to what might be expected based on plasma antibody levels alone.

## Materials and Methods

### Ethics statement

The study design and protocols for the COVID‐19 PROTECT study group were evaluated by National Healthcare Group (NHG) Domain Specific Review Board (DSRB) and approved under study number 2012/00917. Collection of healthy donor samples was approved by SingHealth Centralized Institutional Review Board (CIRB) under study number 2017/2806 and NUS IRB 04‐140. Written informed consent was obtained from participants in accordance with the Declaration of Helsinki for Human Research. The experiments conformed to the principles set out in the Department of Health and Human Services Belmont Report.

### Clinical data and biological sample collection

A total of 55 vaccine breakthrough cases (infected at least 14 days after two doses of vaccine) and 86 of their close contacts (exposed at least 14 days after two doses of vaccine) were recruited into the study from April to June 2021. All vaccine breakthrough cases and close contacts were vaccinated with the Pfizer BNT162b2 vaccine. Demographic data, disease severity, and clinical laboratory data were obtained from patient records throughout hospitalization or during quarantine (Table 1). Blood was collected in Cell Preparation Tubes (CPT) (Becton Dickinson) as soon as possible after infection was confirmed by PCR. Blood samples were also collected from their uninfected close contacts during early time of quarantine. The plasma fraction was extracted for antibody and cytokine analyses, and isolated peripheral blood mononuclear cells (PBMCs) were used for flow cytometry staining for immune cell phenotyping, as well as B cell ELISpot for memory B cell and plasmablast quantification. Antibody and cellular assays were performed subject to sample availability. Plasma samples from a total of 49 patients with primary infection, who were recruited into our earlier cohort from February to August 2020, were also included in our cytokine analysis.

### Serological measurement of SARS‐CoV‐2 antibodies

Plasma specimens (*n* = 55 vaccine breakthrough, *n* = 86 close contact) were stored at −25°C and equilibrated at room temperature before time of analysis. Specimens were analyzed in accordance with the manufacturer’s protocol.

The Elecsys^®^ Anti‐SARS‐CoV‐2 S (Roche S) and Elecsys^®^ Anti‐SARS‐CoV‐2 (Roche N) immunoassays were implemented on the Roche cobas e411 Analyzer (Roche) for the quantitative detection of total antibodies against the SARS‐CoV‐2 spike (S) protein receptor‐binding domain (RBD) and the qualitative detection of total antibodies against the SARS‐CoV‐2 nucleocapsid (N) antigen, respectively. Serum samples were incubated with either a mix of biotinylated and ruthenylated SARS‐CoV‐2 S‐RBD antigens or N antigens, corresponding to the test required, to form immune complexes. The complexes were bound to streptavidin‐coated microparticles upon incubation and then transferred to a measuring cell. For the Roche S assay, the electro‐chemiluminescent signal representing the level of antibodies was measured and samples within the linear range of quantitation (0.4–250 U/ml) were assigned a value. Samples with antibody levels ≥ 0.8 U/ml were considered positive. For the Roche N assay, the cut‐off index (COI) was derived from the measured signal, where samples with COI ≥ 1.0 were considered reactive. The Atellica^®^ IM SARS‐CoV‐2 Total (COV2T) immunoassay was conducted on the Siemens Atellica IM 1600 Analyzer (Siemens Healthcare), for the qualitative detection of total antibodies against the SARS‐CoV‐2 spike 1 RBD recombinant antigen. Preformed complexes of streptavidin‐coated microparticles and biotinylated SARS‐CoV‐2 spike 1 (S1) RBD recombinant antigens were used to capture SARS‐CoV‐2 antibodies within serum samples. Acridinium‐ester‐labeled S1 RBD antigens are then used to detect these bound SARS‐CoV‐2 antibodies. Index values were reported for serum samples, which were considered reactive (≥ 1.00 Index), within a measuring interval of 0.05–10 Index.

### SARS‐CoV‐2 pseudotyped lentivirus production

The pTT5LnX‐CoV‐SP (expressing SARS‐CoV‐2 Spike protein, GenBank: YP_009724390.1, a kind gift from Dr. Brendon John Hanson, DSO National Laboratories) was used as a template plasmid to generate Spike gene of B.1.617.2 Delta variant using QuickChange Lightning Multi Site‐Directed Mutagenesis Kit (Agilent, Cat#210513). Pseudoviruses were generated as previously described (Poh *et al*, 2020) using a third‐generation lentivirus system. Briefly, pseudotyped viral particles expressing SARS‐CoV‐2 Spike proteins were produced by reverse transfection of 293T cells with pTT5LnX‐CoV‐SP (Wuhan WT strain or B.1.617.2 Delta strain), pMDLg/PRRE (a gift from Didier Trono, Addgene #12251), pRSV‐Rev (a gift from Didier Trono, Addgene #12253), and pHIV‐Luc‐ZsGreen (a gift from Bryan Welm, Addgene #39196) and cultured in a 37°C incubator for 3 days. Viral supernatant was harvested, and Lenti‐X p24 rapid titre kit (Takara Bio) was used to quantify the viral titers following the manufacturer’s instructions.

### Pseudovirus neutralization assay

The pseudotyped lentivirus neutralization assay was performed as previously described (Poh *et al*, 2020), with slight modifications. A stable cell line expressing human ACE2, CHO‐ACE2 (a kind gift from Professor Yee‐Joo Tan, Department of Microbiology, NUS & IMCB, A*STAR, Singapore) (Lip *et al*, 2006) was used for the assay. CHO‐ACE2 cells were seeded at 1.8 x 10^4^ per well in a 96‐well black microplate (Corning) in culture medium without Geneticin overnight. Serially diluted heat‐inactivated plasma samples (*n* = 29 vaccine breakthrough, *n* = 86 close contact) at 1:20 to 1:5,120 in fourfold serial dilutions were incubated with equal volume of pseudovirus expressing SARS‐CoV‐2 S proteins of either original wild‐type or Delta mutant strain (5 ng p24 per well) at 37°C for 1 h, before being added to pre‐seeded CHO‐ACE2 cells in duplicate. Cells were refreshed with culture media after 1‐h incubation. After 48 h, cells were washed with PBS and lysed with 1× Passive Lysis Buffer (Promega) with gentle shaking at 125 rpm for 30 min at 37°C. Luciferase activity was subsequently quantified with Luciferase Assay System (Promega) on a GloMax Luminometer (Promega).

### sVNT

Multiplex sVNT was performed using enzymatic biotinylated RBD‐coated MagPlex Avidin microsphere (Luminex). Briefly, RBD‐coated microspheres (600 beads/antigen) were pre‐incubated with diluted plasma (1:400) (*n* = 54 vaccine breakthrough, *n* = 86 close contact) or memory B cell culture supernatant (1:2) (*n* = 48 vaccine breakthrough, *n* = 86 close contact) for 1h at 37°C with 800 rpm agitation. PE‐conjugated ACE2 at 1,000 ng/ml were added into beads followed by 30‐min incubation at 37°C with 800 rpm agitation. Beads were washed twice with 1% BSA‐PBS prior to signal acquisition using MAGPIX Luminex system. Single‐point data were collected.

### Generation of S protein‐expressing cell line

HEK‐293T cells expressing WT SARS‐CoV‐2 S protein were as previously described (Goh *et al*, 2021a, 2021b). For the HEK‐293T cells expressing the Delta variant of SARS‐CoV‐2 S protein, full‐length S gene of the Delta variant was cloned into pHIV‐eGFP transfer plasmid, via the XbaI and BamHI sites, upstream of an IRES (internal ribosome entry site) and an eGFP gene. The transfer plasmid, pHIV‐SARS‐CoV‐2‐DeltaSP‐eGPF, was then co‐transfected with the packaging and envelope plasmids (pMD2.G, pMDLg/pRRE, and pRSV‐Rev) into HEK‐293T cells using EndoFectin Lenti. The medium (DMEM + 10% FBS) was changed 8–16 h later, and the lentiviral particles in the supernatant were collected after a further 48 h of incubation. Cells were transduced by adding the lentiviral supernatant and 8 μg/ml polybrene, then centrifuging at 1,200 *g* for 1 h at room temperature. The medium was changed after 8–16 h in the cell culture incubator. After a further 48 h of incubation, eGFP‐expressing HEK‐293T cells were sorted, expanded, and cryopreserved.

### S protein flow cytometry‐based assay (SFB assay) for antibody detection

The SFB assay was performed as previously described (Goh *et al*, 2021a, 2021b). S protein‐expressing cells were seeded at 1.5 × 10^5^ cells per well in 96‐well V‐bottom plates. The cells were first incubated with human serum (diluted 1:100 in 10% FBS) before a secondary incubation with a double stain, consisting of Alexa Fluor 647‐conjugated anti‐human IgG (diluted 1:500) and propidium iodide (PI; diluted 1:2,500). Cells were read on BD Biosciences LSR4 laser and analyzed using FlowJo (Tree Star). Single‐point data were collected. A sample is defined as positive when the binding is more than mean ± 3SD of the healthy controls (*n* = 22). Plasma samples were run at 1:100 dilution (*n* = 48 vaccine breakthrough, *n* = 86 close contact), while memory B cell supernatants were run at neat dilution (*n* = 48 vaccine breakthrough, *n* = 39 close contact).

### Memory B cell and plasmablast profiling with B cell ELISpot

SARS‐CoV‐2 RBD‐specific plasmablast (*n* = 24 vaccine breakthrough, *n* = 86 close contact) and memory B cell (*n* = 25 vaccine breakthrough, *n* = 86 close contact) numbers were enumerated using the ELISpot Path: Human IgG (SARS‐CoV‐2, RBD) ALP kit (Mabtech), following manufacturer’s instructions. Some participants were not profiled due to lack of sample availability. For plasmablasts, 100,000 or 400,000 fresh PBMCs were resuspended in 200 μl RPMI + 10% FBS and plated in each well of the supplied 96‐well ELISpot plate that was pre‐coated with anti‐IgG capture antibody. The plate was incubated for 18–22 h before washing and addition of RBD‐WASP, followed by anti‐WASP‐ALP, then BCIP/NBT‐plus substrate with development for 2.5–4 min. For memory B cells, 1,000,000 fresh PBMCs were resuspended in 1 ml RPMI + 10% FBS + 1 μg/ml R848 + 10 ng/ml IL‐2 and incubated at 37°C, 5% CO_2_ for 4–6 days to differentiate memory B cells into antibody‐secreting cells. After the incubation, cells were counted, and 100,000 or 400,000 live cells were taken for ELISpot plating as above to enumerate RBD‐specific memory B cells. At the same time, IgG‐secreting cells were also enumerated. To enumerate IgG‐secreting cells, 1,500 or 3,000 live cells were also plated and incubated for 18–22 h before washing and addition of anti‐IgG‐biotin, followed by streptavidin‐ALP, then BCIP/NBT‐plus substrate with development for 1–2.5 min. Following TMB substrate detection, plates were inactivated with 1% Virkon solution for 5 min and then washed and dried overnight. Plates were then read on an IRIS ELISpot reader (Mabtech). Apart from well readings that were saturated, ELISpot readings were calculated based on the average of two wells, and SARS‐CoV‐2 RBD‐specific IgG‐secreting cells were normalized to the total number of IgG‐secreting cells.

### Extracellular and intracellular profiling of T cells with flow cytometry

To profile the SARS‐CoV‐2‐specific T effector subsets in the vaccine breakthrough patients (*n* = 15) and close contacts (*n* = 26), 1,000,000 PBMCs were isolated from peripheral whole blood and rested overnight at 4°C in RPMI 1640 supplemented with 5% human serum, followed by stimulation with pooled SARS‐CoV‐2 PepTivator^®^ S, S1, M, and N peptides (0.6nmol/mL each) (Miltenyi Biotec) for 6 h. Brefeldin A and Monesin (Thermo Fisher Scientific) was added at 2 h post‐stimulation. Cells were stained with surface stain markers in the dark at room temperature for 20 min (Appendix Table S1, no. 1 to 21), followed by fixation and permeabilization for 20 min with Foxp3/ Transcription Factor Staining Buffer Set (Thermo Fisher Scientific). Permeabilized cells were then stained for intracellular cytokines for 20 min (Appendix Table S1, no. 22 to 29). Cells were then acquired with the Cytek^TM^ Aurora cytometer running SpectroFlo^®^ version 2.2.0.3 with automated unmixing. Compensation and analysis of flow cytometry data was performed with FlowJo Version 10.6.1. The gating strategy is shown in Appendix Fig S3.

### Multiplex microbead‐based cytokine immunoassay

Patient plasma samples were inactivated with Triton™ X‐100 (Thermo Fisher Scientific) to a final concentration of 1% for 2 h in the dark. Measurement of immune mediators was done using the Cytokine/Chemokine/Growth Factor 45‐plex Human ProcartaPlex™ (Thermo Fisher Scientific) with the Luminex™ assay (11). Briefly, standards and plasma from vaccine breakthrough patients (*n* = 32), vaccinated close contacts (*n* = 39), primary infection patients (*n* = 49), and healthy controls (*n* = 24) were incubated with fluorescent‐coded magnetic beads pre‐coated with respective antibodies in a black 96‐well clear‐bottom plate overnight at 4°C. After incubation, plates were washed five times with wash buffer (PBS with 1% BSA (Capricorn Scientific) and 0.01% Tween (Promega). Sample‐antibody‐bead complexes were incubated with biotinylated detection antibodies for 1 h and washed five times with wash buffer. Subsequently, streptavidin‐PE was added and incubated for another 30 min. Plates were washed for five times before sample‐antibody‐bead complexes were re‐suspended in sheath fluid for acquisition on the FLEXMAP^®^ 3D (Luminex) using xPONENT^®^ 4.0 (Luminex) software. Data analysis was done on Bio‐Plex Manager^TM^ 6.1.1 (Bio‐Rad). Standard curves were generated with a 5‐PL (5‐parameter logistic) algorithm, reporting values for both mean fluorescence intensity (MFI) and concentration data. The value of Limit of Quantification (LOQ) was assigned to samples with concentrations out of the measurement range.

### Statistical analysis

Statistical analysis was performed using GraphPad Prism 7 software. Unmatched pairwise comparisons were performed using the Mann–Whitney *U*‐test, while matched pairwise comparisons were performed using the Wilcoxon matched‐pairs signed rank test. To compare between multiple groups, Kruskal–Wallis tests and post hoc tests using Dunn’s multiple comparison tests were used to identify significant differences. Spearman’s correlation analyses were performed to calculate correlation coefficient rho and *P* value. *P* < 0.05 were considered significant. ClustVis (Metsalu & Vilo, 2015) was used to compute heat map on the immune mediators. In the heat map presentation, the concentrations of immune mediators were scaled between 0 and 1 for visualization. Principal component analysis (PCA) was performed on the concentrations of immune mediators using Singular Value Decomposition (SVD) method in ClustVis (Metsalu & Vilo, 2015).

## Author contributions

**Matthew Zirui Tay:** Conceptualization; Data curation; Formal analysis; Validation; Investigation; Visualization; Methodology; Writing – original draft; Writing – review & editing. **Angeline Rouers:** Conceptualization; Data curation; Formal analysis; Validation; Investigation; Visualization; Methodology; Writing – review & editing. **Siew‐Wai Fong:** Conceptualization; Investigation; Visualization; Methodology; Writing – review & editing. **Yun Shan Goh:** Supervision; Investigation; Visualization; Methodology; Writing – review & editing. **Yi‐Hao Chan:** Data curation; Validation; Investigation; Methodology; Writing – review & editing. **Zi Wei Chang:** Data curation; Validation; Investigation; Methodology; Writing – review & editing. **Weili Xu:** Data curation; Methodology. **Chee Wah Tan:** Data curation; Investigation; Methodology; Writing – review & editing. **Wan Ni Chia:** Data curation; Investigation; Methodology; Writing – review & editing. **Anthony Torres‐Ruesta:** Data curation; Visualization; Methodology; Writing – review & editing. **Siti Naqiah Amrun:** Data curation; Investigation; Methodology; Project administration; Writing – review & editing. **Yuling Huang:** Investigation; Methodology. **Pei Xiang Hor:** Investigation; Methodology. **Chiew Yee Loh:** Investigation; Methodology. **Nicholas Kim‐Wah Yeo:** Investigation; Methodology; Writing – review & editing. **Bei Wang:** Data curation; Formal analysis; Investigation; Methodology; Writing – review & editing. **Eve Zi Xian Ngoh:** Investigation; Methodology. **Siti Nazihah Mohd Salleh:** Investigation; Methodology. **Jean‐Marc Chavatte:** Data curation; Supervision; Project administration; Writing – review & editing. **Alicia Jieling Lim:** Investigation; Methodology. **Sebastian Maurer‐Stroh:** Resources; Methodology. **Lin‐Fa Wang:** Resources; Supervision; Writing – review & editing. **Raymond, Valentine Tzer Pin Lin:** Resources; Supervision; Project administration. **Cheng‐I Wang:** Resources; Supervision. **Seow‐Yen Tan:** Resources; Supervision; Project administration. **Barnaby, Edwards Young:** Resources; Data curation; Supervision; Project administration; Writing – review & editing. **Yee‐Sin Leo:** Conceptualization; Resources; Supervision; Project administration; Writing – review & editing. **David, C Lye:** Conceptualization; Resources; Supervision; Project administration; Writing – review & editing. **Laurent Renia:** Conceptualization; Resources; Formal analysis; Supervision; Funding acquisition; Project administration; Writing – review & editing. **Lisa, F.P. Ng:** Conceptualization; Resources; Formal analysis; Supervision; Funding acquisition; Project administration; Writing – review & editing.

In addition to the CRediT author contributions listed above, the contributions in detail are:

MZT and AR conceptualized, processed, acquired, analyzed, and interpreted the data and wrote the manuscript. S‐WF and SNA performed the cytokine experiments and analyzed and interpreted the data. YSG, PXH, and CYL performed the SFB assays and analyzed and interpreted the data. Y‐HC, ZWC, WX, NK‐WY, and AT‐R designed and performed the T cell immunophenotyping assays and analyzed and interpreted the data. CWT, WNC, and L‐FW performed the sVNT assay and analyzed and interpreted the data. YH, AR, and MZT performed the memory B cell ELISpot assays and interpreted and analyzed the data. BW, EZXN, SNMS, and CW performed the pseudovirus neutralization assay. J‐MC and AJL performed the Roche and Siemens assays and interpreted and analyzed the data. SM‐S identified and provided the sequence information for the Delta variant used for SFB and pseudovirus neutralization assays. RVTPL, S‐YT, BEY, Y‐SL, and DCL designed and supervised sample collection. LR and LFPN conceptualized, designed, and wrote the manuscript. All authors revised and approved the final version of the manuscript.

## Disclosure and competing interests statement

A patent application for the SFB assay has been filed (Singapore patent #10202009679P: A Method Of Detecting Antibodies And Related Products) by Y.S.G, L.R., and L.F.P.N. The authors declare no other competing interests.

**For more information**

1. World Health Organization (WHO) Coronavirus (COVID‐19) Dashboard: https://covid19.who.int/
2. A*STAR ID Labs website: https://www.a‐star.edu.sg/idlabs

## Supporting information



Appendix

Expanded View Figures PDF

Source Data for Expanded View and Appendix

Source Data for Figure 1

Source Data for Figure 2

Source Data for Figure 3

Source Data for Figure 4

## Data Availability

This study includes no data deposited in external repositories. Source data are provided in the Source Data files. Other data can be obtained upon reasonable request to the corresponding author.
