# Supplementary material for: Decreased memory B cell frequencies in COVID‐19 delta variant vaccine breakthrough infection
Source: EMBO Mol Med. 2022 Jan 21;14(3):e15227. doi: 10.15252/emmm.202115227 (PMC8899913; doi:10.15252/emmm.202115227)
Supplement: Supplementary file 1 — Appendix [file EMMM-14-e15227-s005.pdf]

Appendix Table of Contents

1. Appendix Figure S1
2. Appendix Figure S2
3. Appendix Figure S3
4. Appendix Table S1

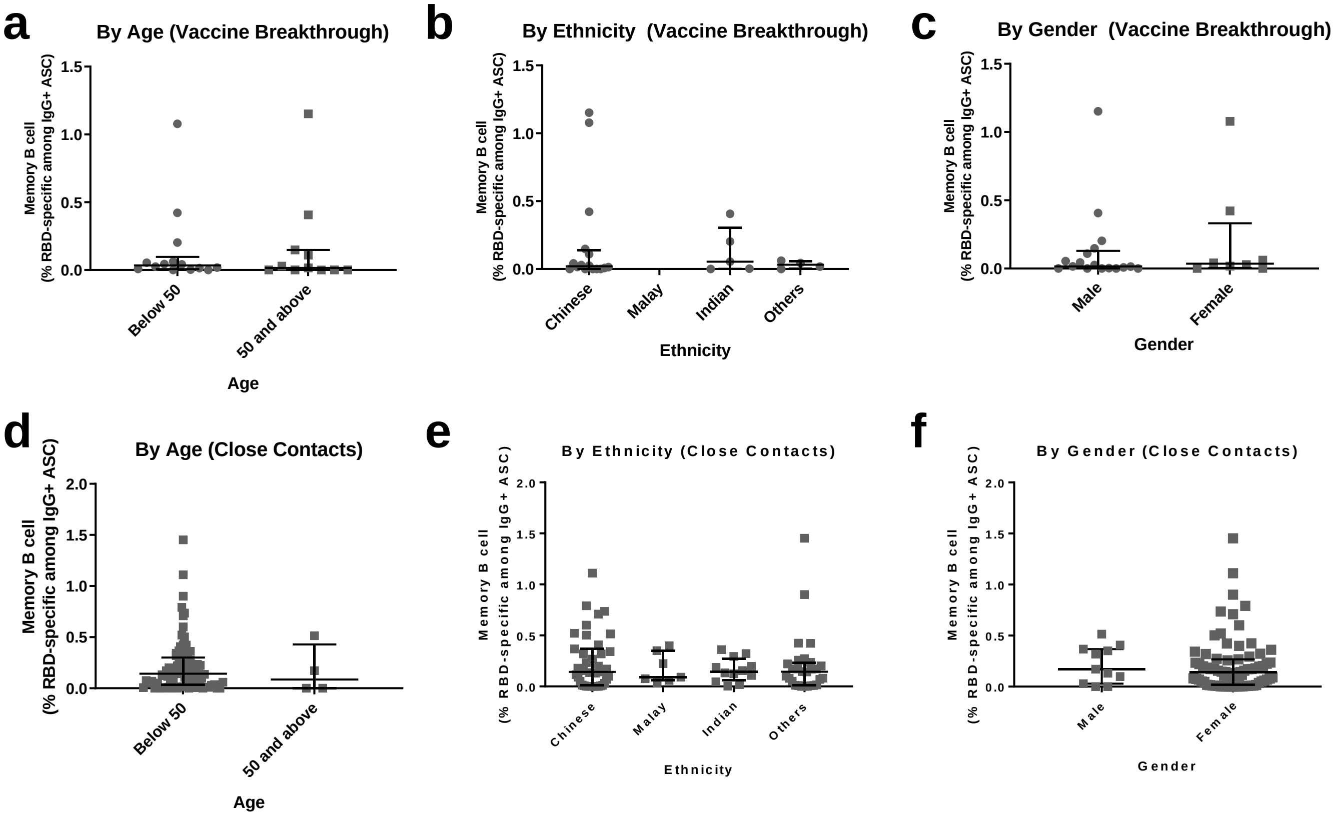

Appendix Figure S1. Memory B cell responses against SARS-CoV-2 are similar across age, ethnicity, and gender. Frequencies of memory B cells specific for SARS-CoV-2 RBD are examined via ELISpot for vaccine breakthrough cases (n=25) and close contacts (n=86). The frequency of RBD-specific memory B cells is given as a percentage of the total IgG-secreting B cell population. Memory B cell frequencies of vaccine breakthrough participants are separated by age (**a**), ethnicity (**b**), or gender (**c**), and close contacts are similarly separated respectively (**d-f**). P values for unpaired comparisons were determined by Mann-Whitney U test or Kruskal-Wallis test with Dunn's multiple corrections tests, but all comparisons showed non-significant results.

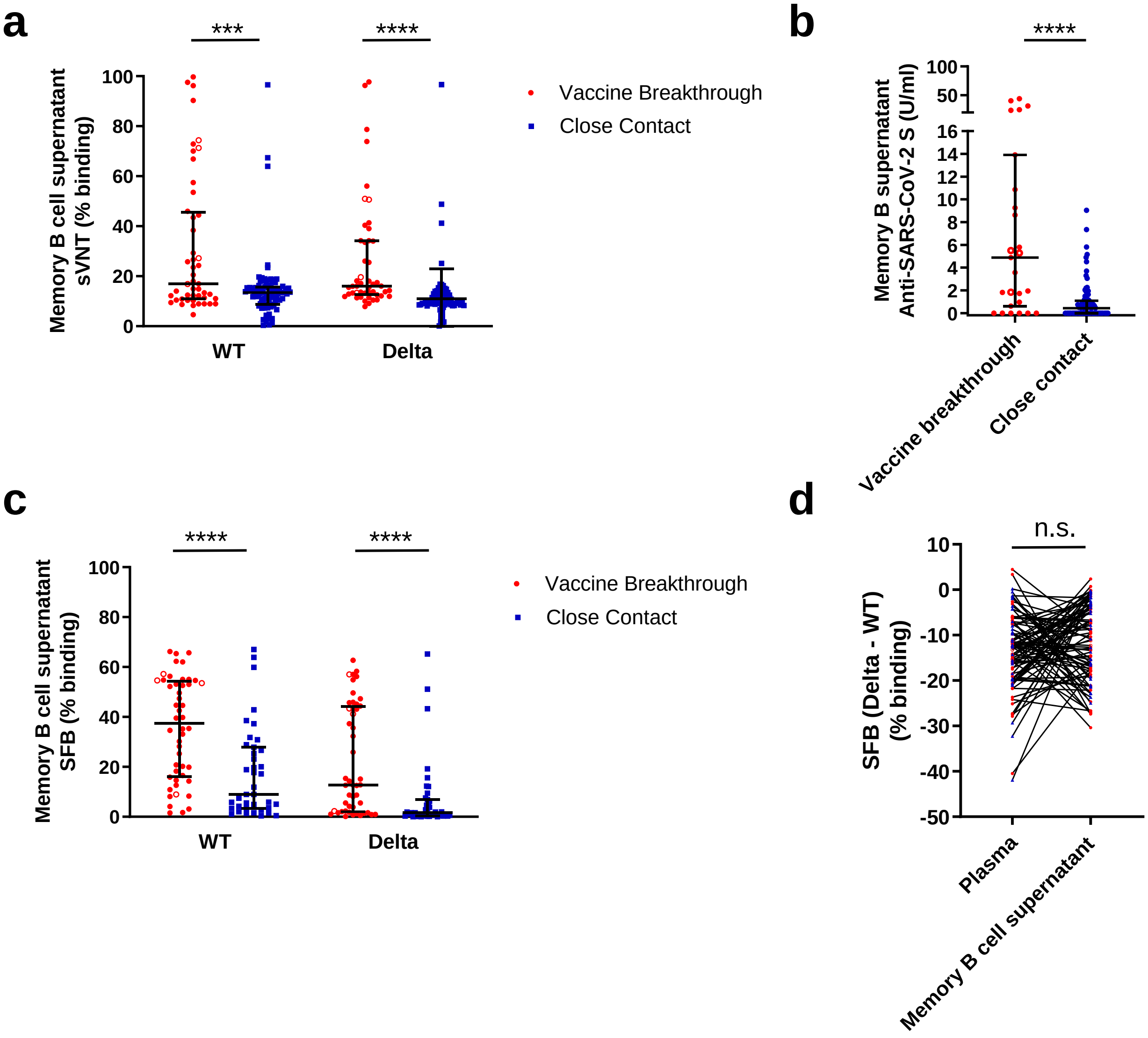

Appendix Figure S2. Memory B cell responses against SARS-CoV-2 in vaccine breakthrough cases and close contacts. a, Levels of anti-SARS-CoV-2 neutralizing antibodies inhibiting ACE2 binding to WT (***: P = 0.0009) or Delta strain (****: P <0.0001) RBD, as determined by surrogate virus neutralization test (sVNT), in supernatant from 5-day culture of activated memory B cells from vaccine breakthrough (n=48) or close contacts (n=86). **b**, Anti-SARS-CoV-2 S protein antibody levels in memory B cell supernatant were determined by Roche Elecsys S antibody assay. **c-d**, Anti-SARS-CoV-2 S protein antibody levels in memory B cell supernatant of vaccine breakthrough cases (n=48) and close contacts (n=39) were determined by SFB. The percentage of plasma antibody-bound cells out of the total population of cells transfected with membrane-anchored SARS-CoV-2 S protein (WT or Delta strain) is given (**c**). The ratio of SFB difference in inhibition between Delta and WT strain is taken, and this ratio is compared between plasma and memory B cell supernatant (**d**). In all graphs error bars denote median and interquartile range. P values for unpaired comparisons were determined by Mann-Whitney U test, and P values for paired comparisons were determined by Wilcoxon matched-pairs signed rank test, **** P<0.0001

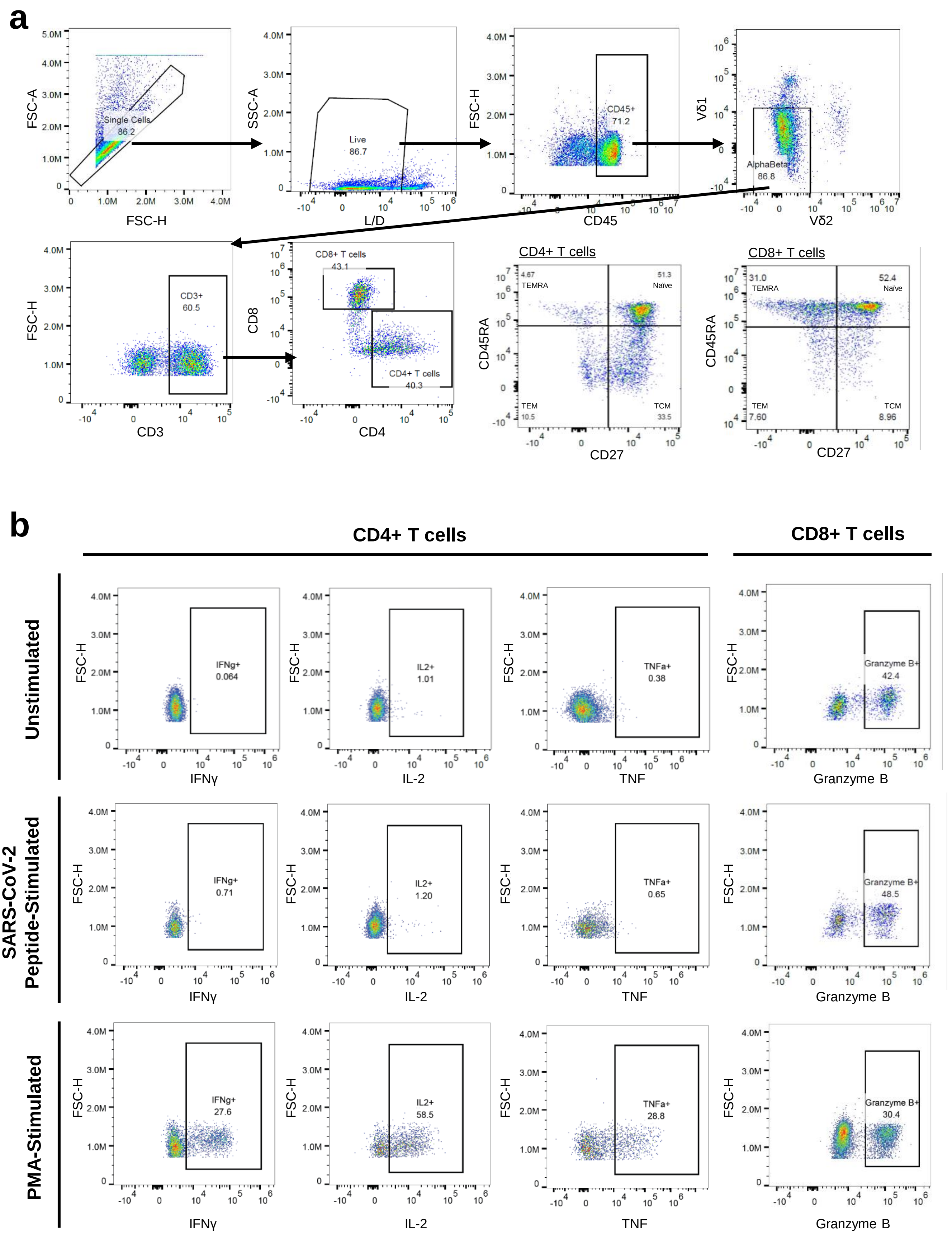

Appendix Figure S3. Gating strategy for CD4+ and CD8+ T cells and intracellular cytokine staining. a, Representative flow cytometry diagrams shown for gating CD4+ and CD8+ T cells, as well as CD27/CD45RA gating for CD4+ and CD8+ T cell differentiation status. **b,** For intracellular cytokine staining, example flow cytometry diagrams from unstimulated (top row), SARS-CoV-2 peptide-stimulated (middle row) and PMA-stimulated (bottom row) conditions are shown.

Appendix Table S1: Flow cytometry antibodies for T cell Panel

No.	Marker	Colour	Volume (μL)	Clone	Cat. No.	Vendor
1	CD66B	BV421	2	G10F5	562940	BD Biosciences
2	CD45RA	SB436	2	H100	62-0458-42	Thermo Fisher
3	CD27	PB	1	O323	302822	Biolegend
4	NKG2C	BV480	2	134591	748168	BD Biosciences
5	CD8	BV605	1	SK1	564116	BD Biosciences
6	CD19	BV605	1	HIB19	740394	BD Biosciences
7	VD2	BV711	1	B6	331412	Biolegend
8	CD107A	BV785	2	H4A3	563869	BD Biosciences
9	NKP46	BB515	2	9-e2	564536	BD Biosciences
10	CD3	SB550	1	SK7	344852	Biolegend
11	CD169	PERCP5.5	5	7-239	346020	Biolegend
12	HLADR	APCR700	2	L243	307626	Biolegend
13	CD4	SN685	1	SK3	344658	Biolegend
14	CD14	APC CY7	1	M0P9	557831	BD Biosciences
15	VD1	APC Vio770	1	REA173	130-120-578	Miltenyi Biotec
16	L/D	ZOMBIE NIR	0.5		423105	Biolegend
17	CXCR5	PE VIO615	1	J252D4	356928	Biolegend
18	CD154	PE CY5	5	TRAP-1	555701	BD Biosciences
19	CD56	PE CY5.5	2	NCAM16.2	35-0567-42	Thermo Fisher
20	CD16	PEAF700	1	3G8	MHCD1624	Thermo Fisher
21	CD45	BUV805	2	H130	612891	BD Biosciences
22	Granzyme B	BV510	2.5	GB11	563388	BD Biosciences
23	IFN-y	BV570	2.5	4S.B3	502534	Biolegend
24	IL-2	BV650	5	MQ1-17H12	564166	BD Biosciences
25	TNF-a	BV750	2.5	MAB11	566359	BD Biosciences
26	IL-6	FITC	2.5	MQ2-13A5	11-7069-82	Thermo Fisher
27	IL-17a	AF647	10	N49-653	560490	BD Biosciences
28	IL-10	PE	2.5	JES-9D7	501404	Biolegend
29	IL-4	PECY7	2.5	8D4-8	560672	BD Biosciences
