# Supplementary material for: Decreased memory B cell frequencies in COVID‐19 delta variant vaccine breakthrough infection
Source: EMBO Mol Med. 2022 Jan 21;14(3):e15227. doi: 10.15252/emmm.202115227 (PMC8899913; doi:10.15252/emmm.202115227)
Supplement: Supplementary file 2 — Expanded View Figures PDF [file EMMM-14-e15227-s007.pdf]

Expanded View Figures

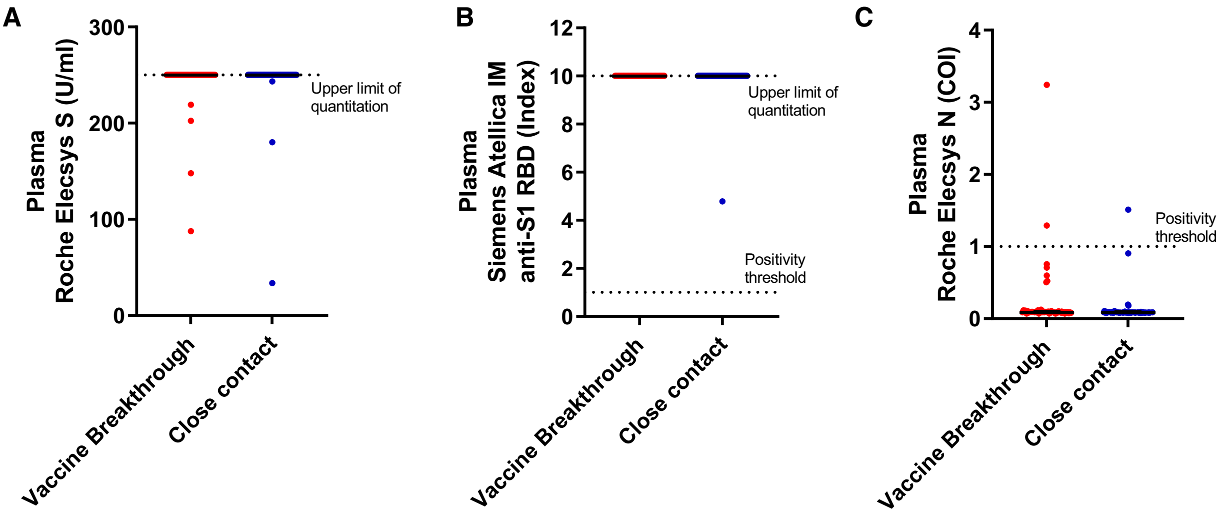

Figure EV1. Anti-SARS-CoV-2 titers in commercial serological assays.

A Anti-SARS-CoV-2 S protein antibodies in plasma of vaccine breakthrough cases (n = 55) or close contacts (n = 86) were determined by Roche Elecsys® S antibody assay. Values above the upper limit of quantitation, 250 U/ml, are truncated to 250 U/ml.

B Anti-SARS-CoV-2 S1 RBD antibodies in plasma of vaccine breakthrough cases (n = 55) or close contacts (n = 86) determined by Siemens Atellica® IM SARS-CoV-2 Total (COVT) assay. Values above the upper limit of quantitation, 10, are truncated to 10.

C Anti-SARS-CoV-2 N protein antibodies in plasma of vaccine breakthrough cases (n = 55) or close contacts (n = 86) determined by Roche Elecsys® N antibody assay.

Data information: Dotted lines indicate upper limit of quantitation (A,B) or positivity threshold (B,C) based on manufacturer's instructions. Error bars indicate median and interquartile range.
Source data are available online for this figure.

© 2022 The Authors

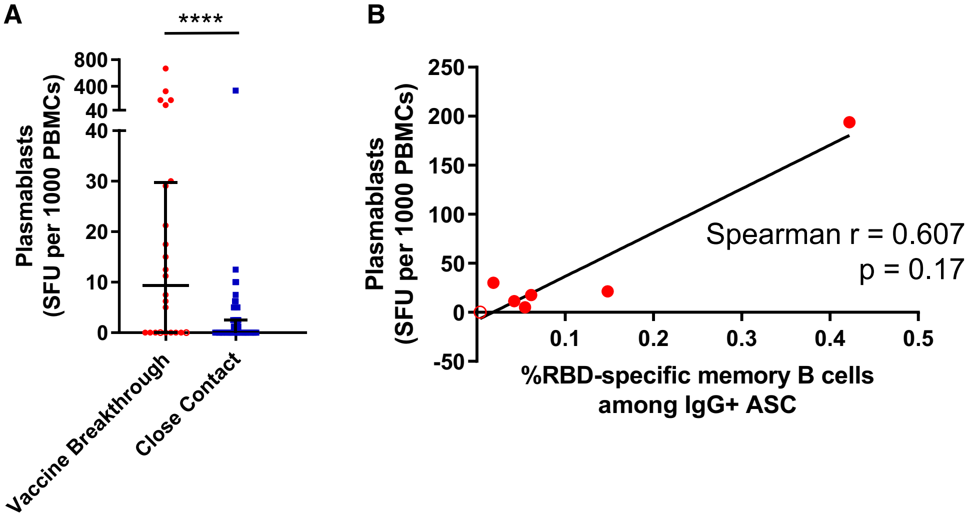

Figure EV2. SARS-CoV-2 RBD-specific plasmablasts are increased in vaccine breakthrough participants.

A Frequencies of plasmablasts specific for SARS-CoV-2 RBD are examined via ELISpot for vaccine breakthrough cases (n = 24) and close contacts (n = 86). The frequency of RBD-specific memory B cells is given as the number of plasmablasts (as determined by spot-forming units, SFU) per 1,000 PBMCs plated. Error bars denote median and interquartile range. P value for unpaired comparison was determined by two-tailed Mann–Whitney U-test, ****$P < 0.0001$.

B The relationship between SARS-CoV-2 RBD-specific plasmablast and memory B cell frequencies in vaccine breakthrough cases was quantified via ELISpot is shown, and correlation was determined by Spearman correlation (n = 7 pairs). Red solid circles indicate Delta strain vaccine breakthrough, and red open circles indicate non-Delta or unknown strain.

Source data are available online for this figure.

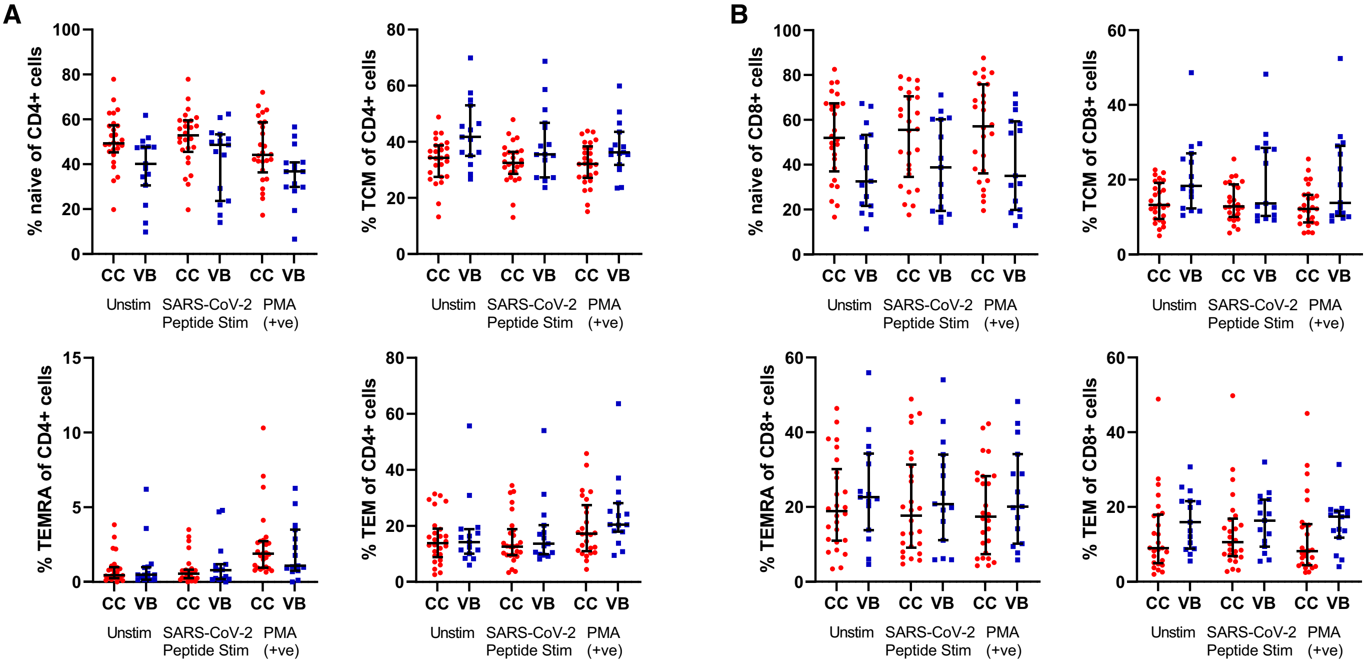

Figure EV3. Differentiation status of T cells by individual.

A, B PBMCs from vaccine breakthrough cases (n = 15) and close contacts (n = 26) were examined for T cell responses. PBMCs were left unstimulated (Unstim), were stimulated with pooled SARS-CoV-2 PepTivator® S, S1, M, and N peptides for 6 h (SARS-CoV-2 Peptide Stim), or non-specifically stimulated with phorbol 12-myristate 13-acetate (PMA), then assessed by high-dimensional flow cytometry. The differentiation status of $CD4^+$ T cells (A) and $CD8^+$ T cells (B) in unstimulated, SARS-CoV-2 peptide-stimulated, and PMA-stimulated conditions were compared based on CD27 and CD45RA expression (Naïve: $CD27^+$ $CD45RA^+$; T central memory (TCM): $CD27^+$ $CD45RA^-$; T effector memory (TEM): $CD27^-$ $CD45RA^-$; TEMRA: $CD27^-$ $CD45RA^+$). Error bars indicate median and interquartile range.

Source data are available online for this figure.

 © 2022 The Authors

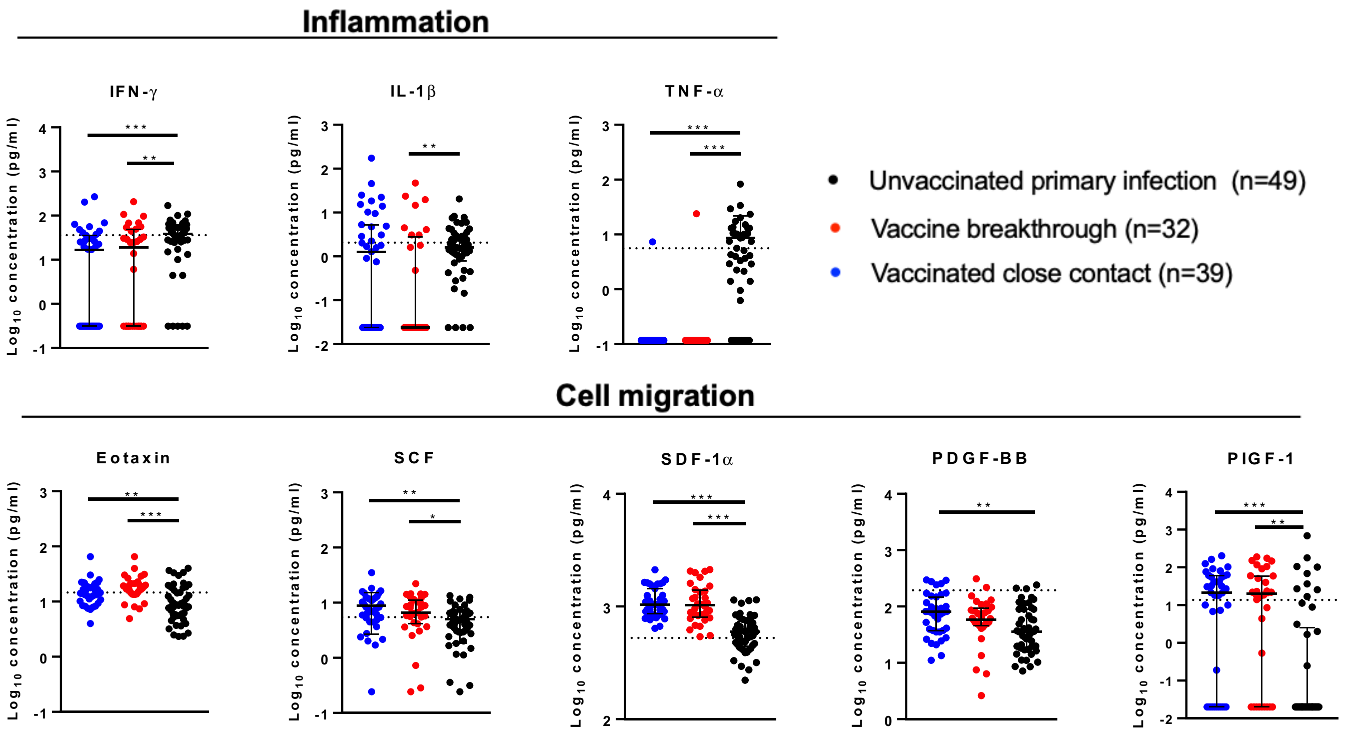

Figure EV4. Cytokine responses in vaccine breakthrough cases and close contacts show milder inflammatory profile compared to primary infection in unvaccinated persons.

Cytokine levels in vaccine breakthrough cases, close contacts, and a matched primary infection cohort were determined by Luminex assay. Selected inflammation-related (top row) and cell migration-related (bottom row) cytokines are shown. Dotted lines represent the average response in a population of healthy controls. In all graphs, error bars denote median and interquartile range. *P* values for unpaired comparisons were determined by two-tailed Mann–Whitney *U*-test, $*P < 0.05$, $**P < 0.01$, $***P < 0.001$.

Source data are available online for this figure.

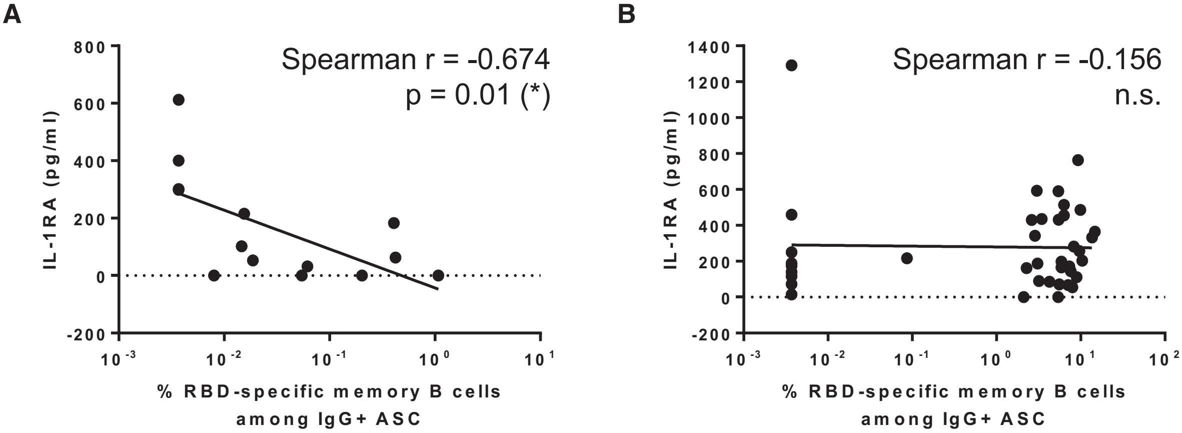

Figure EV5. Correlation between IL-1RA levels and SARS-CoV-2 RBD-specific memory B cells.

A, B The relationship between IL-1RA and log-transformed SARS-CoV-2 RBD-specific memory B cell responses is shown for vaccine breakthrough patients (A) and in their close contacts (B). Dotted lines represent the baseline of 0. Correlation was determined by Spearman correlation, $^{*}P < 0.05$.

Source data are available online for this figure.

 © 2022 The Authors
