# Supplementary material for: Decreased memory B cell frequencies in COVID‐19 delta variant vaccine breakthrough infection
Source: EMBO Mol Med. 2022 Jan 21;14(3):e15227. doi: 10.15252/emmm.202115227 (PMC8899913; doi:10.15252/emmm.202115227)
Supplement: Supplementary file 8 [file EMMM-14-e15227-s004.pdf]

EMBO Molecular Medicine

Decreased memory B cells frequencies in COVID-19 Delta variant vaccine breakthrough infection

Matthew Zirui Tay, Angeline Rouers, Siew-Wai Fong, Yun Shan Goh, Yi-Hao Chan, Zi Wei Chang, Weili Xu, Chee Wah Tan, Wan Ni Chia, Anthony Torres-Ruesta, Siti Naqiah Amrun, Yuling Huang, Pei Xiang Hor, Chiew Yee Loh, Nicholas Kim-Wah Yeo, Bei Wang, Eve Zi Xian Ngoh, Siti Nazihah Mohd Salleh, Jean-Marc Chavatte, Alicia Jieling Lim, Sebastian Maurer-Stroh, Lin-Fa Wang, Raymond Lin, Cheng-I Wang, Seow-Yen Tan, Barnaby Young, Yee-Sin Leo, David Lye, Laurent Renia, and Lisa Ng
DOI: 10.15252/emmm.202115227

Corresponding authors: Lisa Ng (lisa_ng@idlabs.a-star.edu.sg) , Laurent Renia (renia_laurent@IDLabs.a-star.edu.sg)

Review Timeline:

Submission Date:	1st Oct 21
Editorial Decision:	18th Oct 21
Appeal:	21st Oct 21
Editorial Decision:	26th Oct 21
Revision Received:	23rd Nov 21
Editorial Decision:	22nd Dec 21
Revision Received:	23rd Dec 21
Accepted:	23rd Dec 21

Editor: Zeljko Durdevic

Transaction Report:

(Note: With the exception of the correction of typographical or spelling errors that could be a source of ambiguity, letters and reports are not edited. Depending on transfer agreements, referee reports obtained elsewhere may or may not be included in this compilation. Referee reports are anonymous unless the Referee chooses to sign their reports.)

18th Oct 2021

Decision on your manuscript EMM-2021-15227

Dear Dr. Tay,

Thank you for the submission of your manuscript to EMBO Molecular Medicine. We have now received feedback from the two reviewers who agreed to evaluate your manuscript.

As you will see from their reports pasted below, while they recognize interest of your study, they also raise serious concerns, particularly regarding the overall conclusiveness of the presented data. As clear and conclusive insight into a novel, clinically relevant observation is crucial for publication in EMBO Molecular Medicine, and together with the fact that we only accept papers that receive enthusiastic support upon initial review, I am afraid that we cannot offer to consider the manuscript further.

I am sorry that I could not bring better news this time and hope that the referee comments are helpful in your continued work in this area.

Yours sincerely,

Zeljko Durdevic

Zeljko Durdevic
Editor
EMBO Molecular Medicine

***** Reviewer's comments *****

Referee #1 (Comments on Novelty/Model System for Author):

The authors compare patients with active COVID19 with uninfected controls. Thus, the difference in memory B cells is most likely the result of the ongoing infection, rather than the underlying cause of infection (these memory B cells are low, because they have been activated and contribute to the response, likely as plasmablast in tissue). Thus, the experimental design is not appropriate and the conclusions are not supported by the outcomes of this study

Referee #1 (Remarks for Author):

In their manuscript, Zirui Tay et al study antibodies and memory B cells in individuals after vaccination with breakthrough infections of the SARS-CoV-2 delta variant. The topic is extremely timely and interesting as pointe out by the authors, because these infections do occur and it is currently unclear why.
The research question and the technical approached are very clear and appropriate. However, my main concern is the study design. The authors have included patients with a breakthrough infection within 7 days of onset of symptoms. Hence, these are patient with an active infection. These patients are compared to uninfected housemates. Same vaccination status, but not currently experiencing an infection. Thus, the authors compare immune characteristics between infected and uninfected individuals. As a result, any differences are most likely the result of the infection status rather than anything else. I do understand why this was done, but it makes all the outcomes unclear. In fact, it would make sense if there were fewer memory B cells in infected individuals as these should all have been activated and included in the response to infection.
Ideally, this study should be performed in prospectively collected samples from vaccinated individuals before any breakthrough infection and then retrospectively stratified on the basis of a subsequent infection. Then it can be addressed whether pre-existing differences underlie the risk of infection.
Because of the current fault in experimental design, the results do not support the conclusions.

Referee #2 (Comments on Novelty/Model System for Author):

Based on the data provided, I have serious concerns about the technical quality of the flow cytometry data and its analysis and interpretation. The supplementary figures show improper compensation of even easily distinguished surface markers, and the

background subtracted stimulation data suggests that at least some samples exhibited incredibly high levels of background cytokine production (>10%) that point either to issues with the samples or the gating of the flow data.

Referee #2 (Remarks for Author):

Tay et al characterise B cell, T cell and cytokine profiles of a large cohort of COVID-19 vaccine breakthrough infections and close contact controls. The cohort studied is an excellent resource and represents a large number of genuine breakthrough infections with reasonably well-matched controls. Interestingly, the authors find no differences in antibody titres between breakthrough and uninfected vaccinees, suggesting that breakthrough infection did not occur as a result of low neutralising antibody titres after vaccination.

Unfortunately, I feel the authors have missed an opportunity to incisively characterise both pre-existing and de novo T cell responses to various SARS-CoV-2 antigens in this cohort. I have serious concerns regarding the T cell flow cytometry data and its analysis. Appendix Figure S3 shows a partial gating strategy for the identification of CD4 and CD8 T cells, with apparent incorrect unmixing/compensation between CD4 and CD8. The lack of accurate unmixing and lack of representative plots showing any further T cell phenotyping or cytokine production makes it impossible to further assess the flow staining.

Furthermore, the data presented in Figure 3B are very confusing. That a median of 5% of CD8 Tcm cells would produce IL-6 in response to S peptide stimulation several months after vaccination is completely at odds with other reports of the frequency of spike-specific CD8 T cells elicited by vaccination. The presence of some data points at -10% on the graph also indicate that in several samples, the background production of IL-6 in the sample was 10% greater than the stimulated condition, which again points to either issues with the samples (such high spontaneous IL-6 production by T cells in the absence of stimulation is highly unusual) or with the gating and subsequent analysis. It is absolutely critical the authors provide FACS plots to demonstrate the cytokine staining in both stimulated and unstimulated breakthrough and control samples. For reference, what were the frequencies of IFNg+ cells in these samples? Were any positive control (mitogen) stimulations performed?

My other concern lies with the cytokine data in Figure 4. The PCA clearly shows complete overlap of the breakthrough and uninfected control cases, suggesting that the cytokine patterns analysed are not related to ongoing infection. Indeed, the vaccine breakthrough subjects in panel B do not appear to be particularly distinct from the healthy control cases, which are not even shown in the PCA analysis.

21 Oct 2021

Dear Dr Durdevic,

Thank you for your editorial work for our manuscript. We note the decision and would like to request for an appeal regarding our study. We believe that the referees are seriously mistaken in their understanding of the study, and on many occasions did not assess all the data presented. This is very concerning as it appears that the manuscript was not correctly reviewed.

We provide the following as support to explain the misinterpretation of the data. We sincerely hope that you will considering giving us the opportunity to resubmit with a complete point-by-point rebuttal and revised manuscript to further improve the clarity.

<u>Referee #1</u>

Referee #1's major concern is that the lowered memory B cell signal could have been due to activation of the memory B cells upon infection, causing them to differentiate into plasmablasts. However, we wish to explain that our study also measured the plasmablast response in these patients, and our results show that this is not the case (these data were presented in the submitted manuscript).

If the memory B cells were "missing" because they had turned into plasmablasts, we should see higher levels of plasmablasts in the patients with low memory B cells, i.e. the two measure should be anti-correlated. However, we instead observe that memory B cells were positively correlated with plasmablast levels (Fig EV2b).

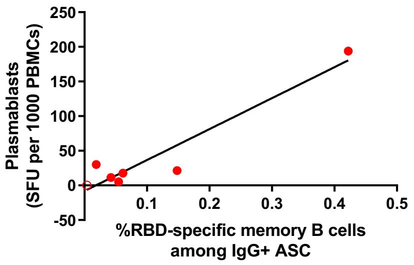

Thus, while we are in full agreement that we are here comparing infected individuals with uninfected individuals. We disagree that our main conclusions, including the surprising finding for memory B cells, are affected by this.

Notably, Referee #2 is in agreement with us here, noting that "the cohort studied in an excellent resource and represents a large number of genuine breakthrough infections with reasonably well-matched controls".

<u>Referee #2</u>

Referee #2's main concern was regarding the technical approach on the T cell analysis.

1. Referee #2 was concerned that there was "apparent incorrect unmixing/compensation".

   We wish to explain that a spectral flow cytometer was used here and standard automated unmixing analysis utilized. We are happy to provide representative plots showing further example flow cytometry plots, attached here in the Annex (Annex – Flow Cytometry Back-Gating).

As is clear from the plots, there is largely no drift of datapoints – and where some drift exists, it does not cause signal spillover across groups.

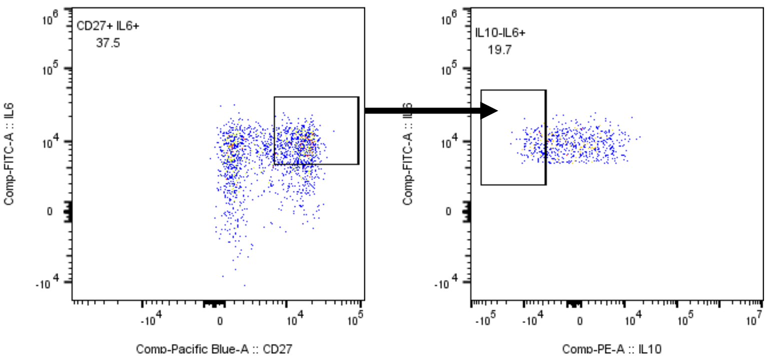

2. Referee #2 had concerns with Fig. 3, noting that the percentages observed by us were higher than expected.

   This concern is primarily due to a difference in interpretation for Fig. 3. In Fig. 3, the Phenographs were assigned by automatic clustering, not traditional gating methods. Labels were only given afterwards to describe the cluster phenotype based on the MFI for each marker. This differs from typical gating strategies, where only a small highly distinct population is labelled positive. This was necessary to provide a qualitative description of the Phenograph clusters. This accounts for the larger percentages that we observe. To help clarify the populations in view, we have here attached flow cytometry plots for with gating for each cluster, based on the Phenograph cluster's MFI in the highlighted parameters (Annex – Flow Cytometry Back-Gating).

<u>CD8 Cluster 13</u>

Close Contact (PTID64)- Peptide stimulated

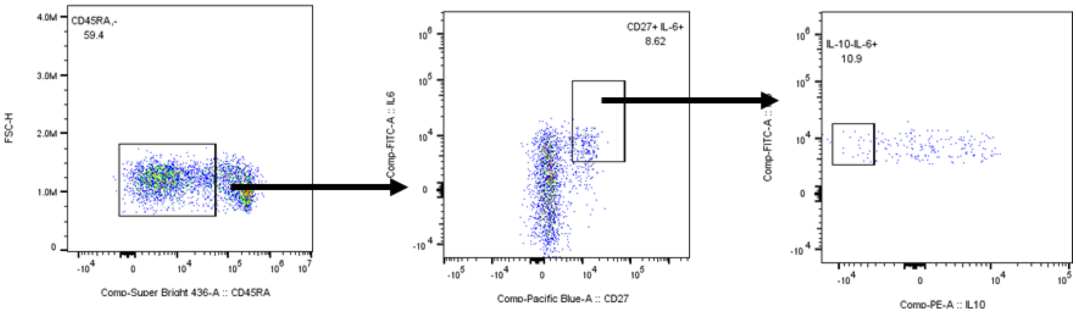

   However, it should be noted that these gatings are approximate only, since it is impossible to completely replicate the automated multiparameter gating strategy decided by the Phenograph clustering algorithm. For each cluster, we have presented representative flow cytometry plots from one vaccine breakthrough and one close contact case respectively to showcase the differences observed.

   Because the same number of cells were taken for each patient, the large negative percentages in Fig. 3c may not be due to an absolute decrease in that population, but a relative decrease due to increase in other populations. Indeed, the lowest datapoint in Fig. 3c (-12.3%) corresponds to the highest datapoint in Fig. 3b (+15.3%), and the second-lowest datapoint in Fig 3c (-4.7%) corresponds to the third highest datapoint in Fig. 3b (+7.8%). To further clarify this, we have also now modified the source data to show both the percentages of cells in each Phenograph cluster for stimulated and unstimulated conditions separately, attached in the updated Source Data for Figure 3.

   Positive mitogen controls were performed for all samples (stimulating with phorbol myristate acetate (PMA)), but were not presented in the manuscript since we did not

think that they were physiologically relevant. We have now added a representative set of plots showing PMA stimulation, attached in the Annex (Annex – Flow Cytometry Back-Gating).

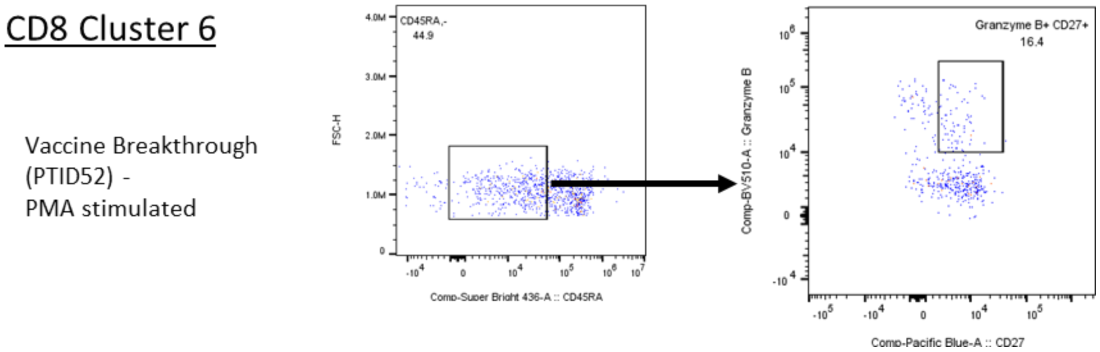

3. Referee #2 had concerns with the cytokine data in Fig. 4, noting that “the vaccine breakthrough subjects do not appear to be particularly distinct from the healthy control cases”.

   We believe that the reviewer misunderstood the figure. Actually, this is precisely our point here: vaccination is protective to such a degree that even despite vaccine breakthrough, the inflammatory cytokine profile of these individuals remains largely similar to healthy, uninfected individuals.

   This was true across the comprehensive array of cytokines that were analysed in this dataset (See Figure 4 Source Data for full list).

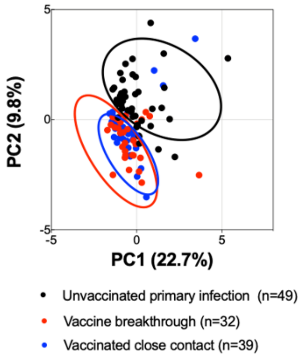

Notably, Referee #1 is in agreement with us here, since he was pleased with the technical approach, commenting that “the technical approaches are very clear and appropriate”.

We hope that these clarifications will set right the reviewers’ misunderstandings and provide further substantiation for our study and its high-impact conclusions.

Best regards,

Lisa F.P. Ng, PhD

Executive Director, A*STAR Infectious Diseases Labs

26th Oct 2021

Dear Dr. Tay,

Thank you for your response to the editorial decision on your manuscript entitled "Decreased memory B cells frequencies in COVID-19 Delta variant vaccine breakthrough infection". I have now carefully examined the arguments provided in your letter and discussed them with the other members of our editorial team. Additionally, I have sought external advice on the study from an expert in the field.

I am pleased to inform you that we decided to re-consider our initial decision and to invite major revision of your manuscript. Please provide detailed responses to the referee concerns and appropriately amend the manuscript to strengthen main message of the study.

Further consideration of a revision that addresses reviewers' concerns in full will entail a second round of review. EMBO Molecular Medicine encourages a single round of revision only and therefore, acceptance or rejection of the manuscript will depend on the completeness of your responses included in the next, final version of the manuscript. For this reason, and to save you from any frustrations in the end, I would strongly advise against returning an incomplete revision.

We would welcome the submission of a revised version within three months for further consideration. Please let us know if you require longer to complete the revision.

I look forward to receiving your revised manuscript.Please use this link to login to the manuscript system and submit your revision: https://embomolmed.msubmit.net/cgi-bin/main.plex

Yours sincerely,

Zeljko Durdevic

Zeljko Durdevic
Editor
EMBO Molecular Medicine

Referee #1 (Comments on Novelty/Model System for Author):

The authors compare patients with active COVID19 with uninfected controls. Thus, the difference in memory B cells is most likely the result of the ongoing infection, rather than the underlying cause of infection (these memory B cells are low, because they have been activated and contribute to the response, likely as plasmablast in tissue). Thus, the experimental design is not appropriate and the conclusions are not supported by the outcomes of this study.

Referee #1 (Remarks for Author):

In their manuscript, Zirui Tay et al study antibodies and memory B cells in individuals after vaccination with breakthrough infections of the SARS-CoV-2 delta variant. The topic is extremely timely and interesting as pointe out by the authors, because these infections do occur and it is currently unclear why.

The research question and the technical approached are very clear and appropriate. However, my main concern is the study design. The authors have included patients with a breakthrough infection within 7 days of onset of symptoms. Hence, these are patient with an active infection. These patients are compared to uninfected housemates. Same vaccination status, but not currently experiencing an infection. Thus, the authors compare immune characteristics between infected and uninfected individuals. As a result, any differences are most likely the result of the infection status rather than anything else. I do understand why this was done, but it makes all the outcomes unclear. In fact, it would make sense if there were fewer memory B cells in infected individuals as these should all have been activated and included in the response to infection.

Ideally, this study should be performed in prospectively collected samples from vaccinated individuals before any breakthrough infection and then retrospectively stratified on the basis of a subsequent infection. Then it can be addressed whether pre-existing differences underlie the risk of infection.

Because of the current fault in experimental design, the results do not support the conclusions.

Authors' Responses to Referee #1

- *"In their manuscript, Zirui Tay et al study antibodies and memory B cells in individuals after vaccination with breakthrough infections of the SARS-CoV-2 delta variant. The topic is extremely timely and interesting as pointe out by the authors, because these infections do occur and it is currently unclear why."*

Response: We thank Referee #1 for his/her favourable evaluation of the importance of the paper topic.

- *"The research question and the technical approached are very clear and appropriate."*

Response: We thank Referee #1 for his/her favourable evaluation of our methodological approaches and their clarity.

- *"… the authors compare immune characteristics between infected and uninfected individuals."*

“Thus, the difference in memory B cells is most likely the result of the ongoing infection, rather than the underlying cause of infection (these memory B cells are low, because they have been activated and contribute to the response, likely as plasmablast in tissue).”

Response: We thank Referee #1 for the critical examination of our paper. However, we respectfully disagree with Referee #1’s conclusion that the difference in memory B cells is the result of the ongoing infection, and that the lowered memory B cell signal could have been due to activation of the memory B cells upon infection, causing them to differentiate into plasmablasts. Notably, we measured the plasmablast response in these patients, and our results show that this was not the case. If the memory B cells were “missing” because they had turned into plasmablasts, we should observe higher levels of plasmablasts in the patients with low memory B cells, i.e. the two measures should be anti-correlated. However, we instead observe that memory B cells were positively correlated with plasmablast levels (Fig EV2b). Thus, while we are in full agreement that we are here comparing infected individuals with uninfected individuals, we disagree that our main conclusions, including the surprising finding for memory B cells, are affected by this. We have now inserted text into the manuscript (lines 168-171) to clarify this point.

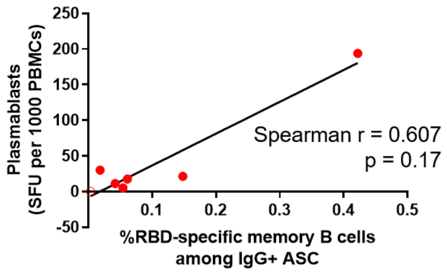

- *Ideally, this study should be performed in prospectively collected samples from vaccinated individuals before any breakthrough infection and then retrospectively stratified on the basis of a subsequent infection.*

Response: We agree that a prospective study would provide important confirmation of the findings here. However, such a study is much more logistically challenging, especially at the scale of the number of patients analysed here. Perhaps due to this reason, previous prospective studies have focused on plasma antibody responses since plasma is much easier to process and store as compared to PBMCs. Our study is thus uniquely positioned in evaluating B and T cell responses in addition to plasma antibody responses in the context of vaccine breakthrough infection.

Referee #2 (Comments on Novelty/Model System for Author):

Based on the data provided, I have serious concerns about the technical quality of the flow cytometry data and its analysis and interpretation. The supplementary figures show improper compensation of even easily distinguished surface markers, and the background subtracted stimulation data suggests that at least some samples exhibited incredibly high levels of background cytokine production (>10%) that point either to issues with the samples or the gating of the flow data.

Referee #2 (Remarks for Author):

Tay et al characterise B cell, T cell and cytokine profiles of a large cohort of COVID-19 vaccine breakthrough infections and close contact controls. The cohort studied is an excellent resource and represents a large number of genuine breakthrough infections with reasonably well-matched controls. Interestingly, the authors find no differences in antibody titres between breakthrough and uninfected vaccinees, suggesting that breakthrough infection did not occur as a result of low neutralising antibody titres after vaccination.

Unfortunately, I feel the authors have missed an opportunity to incisively characterise both pre-existing and de novo T cell responses to various SARS-CoV-2 antigens in this cohort. I have serious concerns regarding the T cell flow cytometry data and its analysis. Appendix Figure S3 shows a partial gating strategy for the identification of CD4 and CD8 T cells, with apparent incorrect unmixing/compensation between CD4 and CD8. The lack of accurate unmixing and lack of representative plots showing any further T cell phenotyping or cytokine production makes it impossible to further assess the flow staining.

Furthermore, the data presented in Figure 3B are very confusing. That a median of 5% of CD8 Tcm cells would produce IL-6 in response to S peptide stimulation several months after vaccination is completely at odds with other reports of the frequency of spike-specific CD8 T cells elicited by vaccination. The presence of some data points at -10% on the graph also indicate that in several samples, the background production of IL-6 in the sample was 10% greater than the stimulated condition, which again points to either issues with the samples (such high spontaneous IL-6 production by T cells in the absence of stimulation is highly unusual) or with the gating and subsequent analysis. It is absolutely critical the authors provide FACS plots to demonstrate the cytokine staining in both stimulated and unstimulated breakthrough and control samples. For reference, what were the frequencies of IFNg+ cells in these samples? Were any positive control (mitogen) stimulations performed?

My other concern lies with the cytokine data in Figure 4. The PCA clearly shows complete overlap of the breakthrough and uninfected control cases, suggesting that the cytokine patterns analysed are not related to ongoing infection. Indeed, the vaccine breakthrough subjects in panel B do not appear to be particularly distinct from the healthy control cases, which are not even shown in the PCA analysis.

Authors' Responses to Referee #2

- *Tay et al characterise B cell, T cell and cytokine profiles of a large cohort of COVID-19 vaccine breakthrough infections and close contact controls. The cohort studied is an excellent resource and represents a large number of genuine breakthrough infections with reasonably well-matched controls.*

Response: We thank Referee #2 for the favourable evaluation of the usefulness and quality of our cohort study.

- *Unfortunately, I feel the authors have missed an opportunity to incisively characterise both pre-existing and de novo T cell responses to various SARS-CoV-2 antigens in this cohort.*

Response: Unfortunately, we did not examine the differences in T cell responses to the different SARS-CoV-2 antigens in this cohort, since a pooled set of SARS-CoV-2 peptides (S, S1, M and N peptides) was used rather than separate antigens. In the antibody response, very few vaccine breakthrough cases (2/55) showed (presumably *de novo*) responses to the N protein, and it would be interesting to see if this was similar in the T cell compartment. Unfortunately, we are unable to perform this experiment since the PBMC samples from these patients are no longer available.

- *I have serious concerns regarding the T cell flow cytometry data and its analysis. Appendix Figure S3 shows a partial gating strategy for the identification of CD4 and CD8 T cells, with apparent incorrect unmixing/compensation between CD4 and CD8. The lack of accurate unmixing and lack of representative plots showing any further T cell phenotyping or cytokine production makes it impossible to further assess the flow staining.*

Response: We thank the reviewer for highlighting this where in the original Appendix Fig S3, the CD8 was undercompensated relative to CD4. We had initially accepted this since the CD4+ and CD8+ T cell populations were distinct regardless. However, we took the comments serious and we have gone over our compensations and adjusted them accordingly. We have also now revised Appendix Fig S3, such that Appendix S3a shows the full gating strategy for all T cell phenotyping, and Appendix Fig S3b shows cytokine production in unstimulated, SARS-CoV-2 peptide-stimulated, and positive control PMA-stimulated conditions.

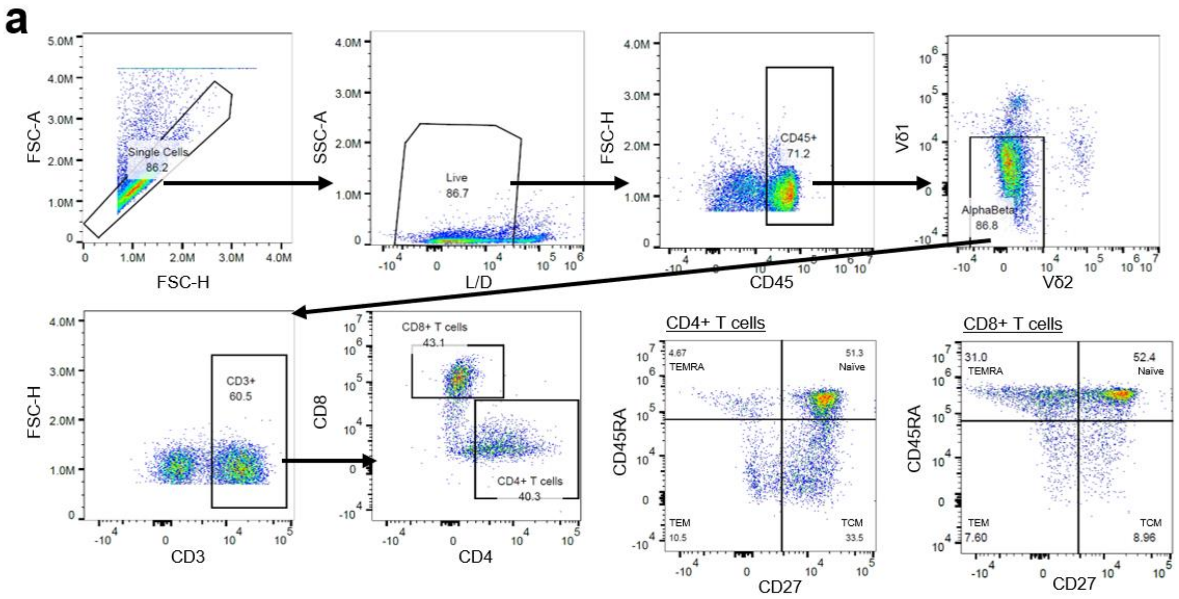

Appendix Figure S3a. Gating strategy for CD4+ and CD8+ T cells and intracellular cytokine staining. *Representative flow cytometry diagrams shown for gating CD4+ and CD8+ T cells, as well as CD27/CD45RA gating for CD4+ and CD8+ T cell differentiation status.*

- *It is absolutely critical the authors provide FACS plots to demonstrate the cytokine staining in both stimulated and unstimulated breakthrough and control samples.*

Response: We thank the reviewer for this suggestion and we have now included the flow cytometry diagrams for the cytokine staining for unstimulated, peptide-stimulated, and PMA-stimulated conditions in Appendix Fig S3b.

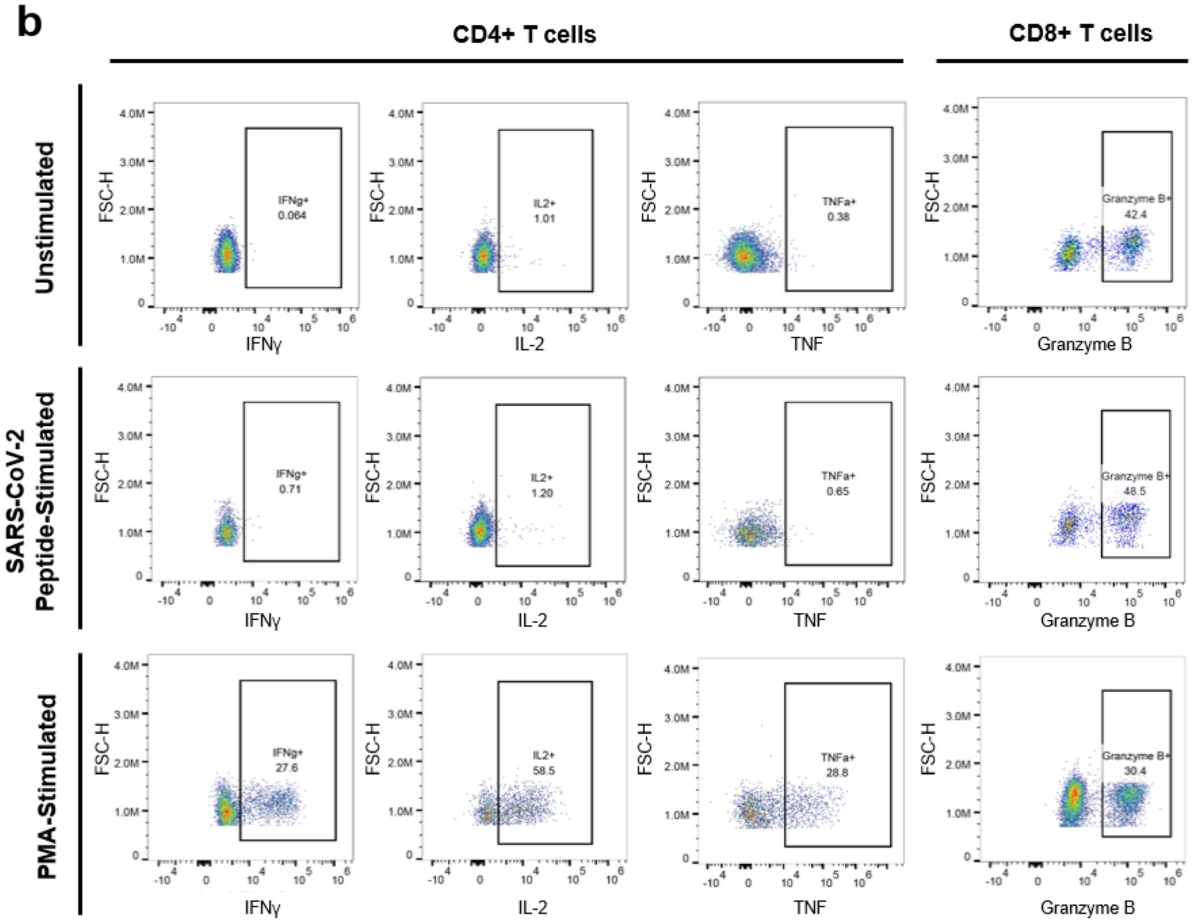

Appendix Figure S3b. Gating strategy for CD4+ and CD8+ T cells and intracellular cytokine staining. *Representative flow cytometry diagrams shown for gating CD4+ and CD8+ T cells for intracellular cytokine staining, example flow cytometry diagrams from unstimulated (top row), SARS-CoV-2 peptide-stimulated (middle row) and PMA-stimulated (bottom row) conditions are shown.*

- *Furthermore, the data presented in Figure 3B are very confusing. That a median of 5% of CD8 Tcm cells would produce IL-6 in response to S peptide stimulation several months after vaccination is completely at odds with other reports of the frequency of spike-specific CD8 T cells elicited by vaccination.*

Response: We note this comment and wish to explain that this confusion was caused by the original T cell analysis. The original approach assigned clusters that were labelled based on their relative fluorescence intensity, leading to much less stringent gating cut-offs than typically used for assigning positivity. This led to elevated subpopulation frequencies. Upon further consideration, we have decided to remove the dimensionality reduction and clustering approach for the T cell analysis, and have opted for a traditional gating, highlighting common cytokine/effector molecules and identifying polyfunctionality, since these are common T cell measures that have also been published in other recent papers on the T cell response against SARS-CoV-2 in vaccination and infection settings (Breton *et al*, 2021; Guerrera *et al*, 2021; Tan *et al*, 2021). These modifications are in lines 182-198 and lines 250-257. We sincerely believe that with the modified figures, this now removes unnecessary complexity and better streamlines the paper.

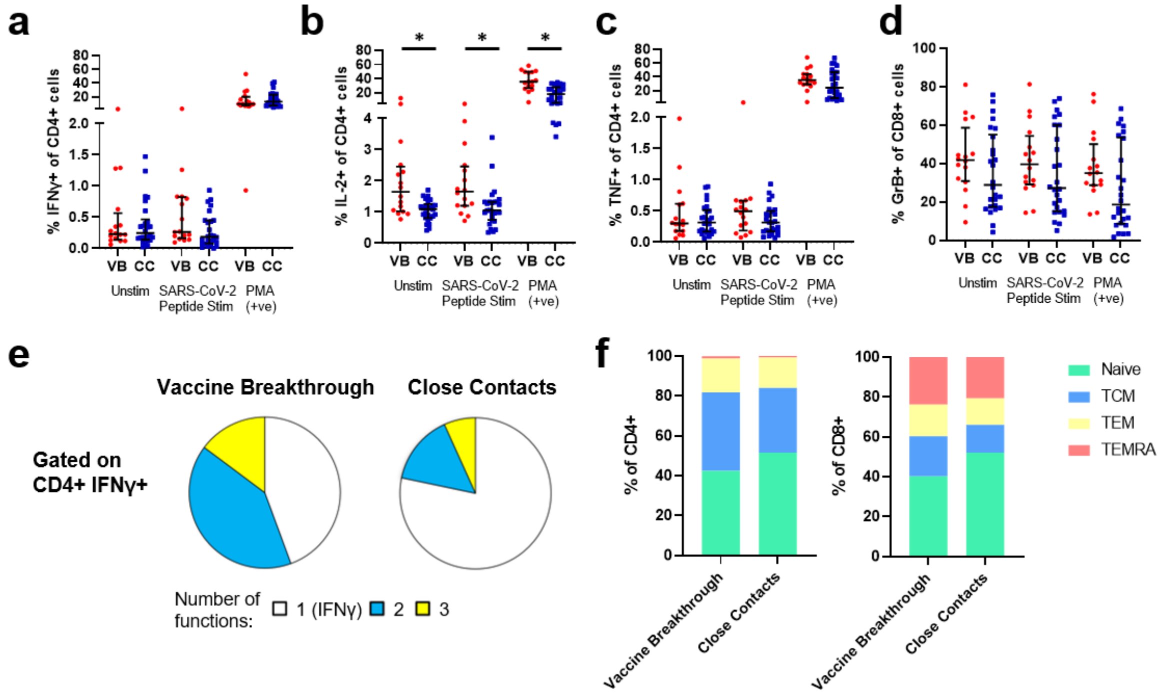

Figure 3. T cell responses in vaccine breakthrough cases and close contacts. a-d, *PBMCs from vaccine breakthrough cases (n=16) and close contacts (n=26) were examined for T cell responses. PBMCs were left unstimulated (Unstim), were stimulated with pooled SARS-CoV-2 PepTivator® S, S1, M and N peptides for 6 hours (SARS-CoV-2 Peptide Stim), or non-specifically stimulated with phorbol 12-myristate 13-acetate (PMA), then assessed by high-dimensional flow cytometry. CD4+ T cells positive for intracellular staining of IFNγ (a), IL-2 (b), TNF (c), or CD8+ T cells positive for intracellular staining of granzyme B (d) are shown.* ***e****, To examine polyfunctionality of CD4+ T cells, SARS-CoV-2 peptide-stimulated IFNγ+ CD4+ T cells were further examined for co-expression of IL-2 and/or TNF, and the fraction of cells expressing IFNγ only (1 function),*

IFNγ and either IL-2 or TNF (2 functions), or IFNγ and both IL-2 and TNF (3 functions) are shown. ***f****, The differentiation status of SARS-CoV-2 peptide-stimulated CD4+ T cells (left) and CD8+ T cells (right) were compared based on CD27 and CD45RA expression (Naïve: CD27+ CD45RA+; T central memory (TCM): CD27+ CD45RA-; T effector memory (TEM): CD27- CD45RA-; TEMRA: CD27- CD45RA+). The average of all vaccine breakthrough cases or close contacts are plotted respectively. P value for unpaired comparison was determined by two-tailed Mann-Whitney U test, ** P<0.01, **** P<0.0001*

- Were any positive control (mitogen) stimulations performed?

Response: Yes, stimulation with phorbol 12-myristate 13-acetate (PMA) was performed in parallel as a positive control. We have now included the data for unstimulated controls and PMA-stimulated controls in Fig 3a-d and Fig EV3.

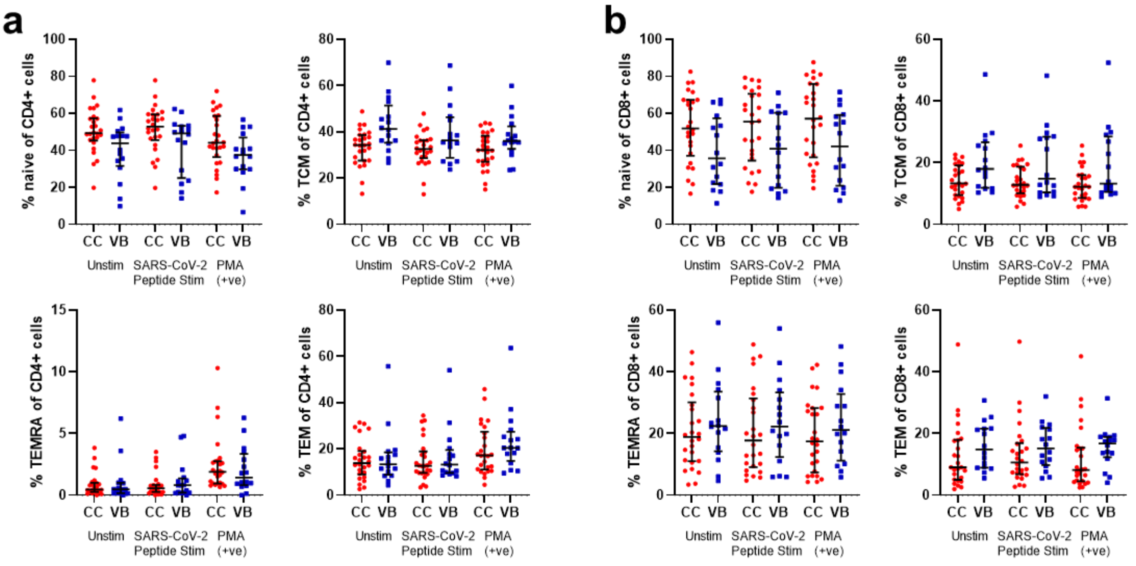

Figure EV3. Differentiation status of T cells by individual. *The differentiation status of CD4+ T cells (a) and CD8+ T cells (b) in unstimulated, SARS-CoV-2 peptide-stimulated, and PMA-stimulated conditions were compared based on CD27 and CD45RA expression (Naïve: CD27+ CD45RA+; T central memory (TCM): CD27+ CD45RA-; T effector memory (TEM): CD27- CD45RA-; TEMRA: CD27- CD45RA+).*

- For reference, what were the frequencies of IFNg+ cells in these samples?

Response: Vaccine breakthrough patients showed a median of 0.26% IFNγ+ CD4+ T cells (interquartile range: 0.19%-0.81%) after peptide stimulation, and close contacts showed a median of 0.19% IFNγ+ CD4+ T cells (interquartile range: 0.08%-0.45%) after peptide stimulation. These values are in approximate agreement with the frequencies of IFNγ+ CD4+ T cells found after BNT162b2 vaccination (Guerrera *et al.*, 2021). This data is now also shown in the revised Fig 3a.

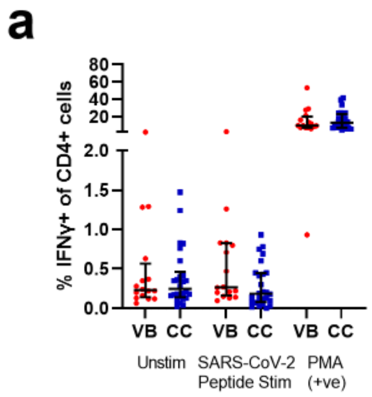

Figure 3a. T cell IFNγ responses in vaccine breakthrough cases and close contacts. *PBMCs from vaccine breakthrough cases (n=16) and close contacts (n=26) were examined for T cell responses. PBMCs were left unstimulated (Unstim), were stimulated with pooled SARS-CoV-2 PepTivator® S, S1, M and N peptides for 6 hours (SARS-CoV-2 Peptide Stim), or non-specifically stimulated with phorbol 12-myristate 13-acetate (PMA), then assessed by high-dimensional flow cytometry. CD4+ T cells positive for intracellular staining of IFNγ are shown.*

- *My other concern lies with the cytokine data in Figure 4. The PCA clearly shows complete overlap of the breakthrough and uninfected control cases, suggesting that the cytokine patterns analysed are not related to ongoing infection. Indeed, the vaccine breakthrough subjects in panel B do not appear to be particularly distinct from the healthy control cases, which are not even shown in the PCA analysis.*

Response: We note Referee #2's surprise at the results, and indeed, the similarity between vaccine breakthrough subjects and uninfected controls is precisely the interesting point here: vaccination appears to be protective to such a degree that even despite vaccine breakthrough, the inflammatory cytokine profile of these individuals remains largely similar to uninfected individuals. This was the result from analysis of a comprehensive panel of 45 cytokines were included for the PCA, common infection and inflammation-related cytokines such as IL-1β, TNF, IFNγ, MIP-1α, RANTES, and CXCL10 (See Figure 4 Source Data for full list). We have now also included the unvaccinated healthy controls in the PCA as well. Interestingly, the unvaccinated healthy controls cluster away from both the vaccinated group and primary infection group. The differences from the vaccinated group may be due to effects of the vaccine on innate immunity in addition to adaptive immunity, as has been reported by other groups (Arunachalam *et al*, 2021; Föhse *et al*, 2021). Nevertheless, we chose not to emphasize this in the paper as the comparison of vaccinated vs healthy unvaccinated groups is not the primary focus of the paper.

References

Arunachalam PS, Scott MKD, Hagan T, Li C, Feng Y, Wimmers F, Grigoryan L, Trisal M, Edara VV, Lai L *et al* (2021) Systems vaccinology of the BNT162b2 mRNA vaccine in humans. *Nature* 596: 410-416

Breton G, Mendoza P, Hägglöf T, Oliveira TY, Schaefer-Babajew D, Gaebler C, Turroja M, Hurley A, Caskey M, Nussenzweig MC (2021) Persistent cellular immunity to SARS-CoV-2 infection. *The Journal of experimental medicine* 218

Föhse FK, Geckin B, Overheul GJ, van de Maat J, Kilic G, Bulut O, Dijkstra H, Lemmers H, Sarlea SA, Reijnders M *et al* (2021) The BNT162b2 mRNA vaccine against SARS-CoV-2 reprograms both adaptive and innate immune responses. *medRxiv*: 2021.2005.2003.21256520

Guerrera G, Picozza M, D'Orso S, Placido R, Pirronello M, Verdiani A, Termine A, Fabrizio C, Giannessi F, Sambucci M *et al* (2021) BNT162b2 vaccination induces durable SARS-CoV-2 specific T cells with a stem cell memory phenotype. *Sci Immunol*: eabl5344

Tan AT, Linster M, Tan CW, Le Bert N, Chia WN, Kunasegaran K, Zhuang Y, Tham CYL, Chia A, Smith GJD *et al* (2021) Early induction of functional SARS-CoV-2-specific T cells associates with rapid viral clearance and mild disease in COVID-19 patients. *Cell Rep* 34: 108728

22nd Dec 2021

Dear Dr. Ng,

Thank you for the submission of your revised manuscript to EMBO Molecular Medicine. We have now heard back from a referee who agreed to re-evaluate your manuscript. In addition, I have also sought external advice on the study from an expert in the field. As you will see from the report below, the referee #2 appreciates your efforts in addressing the referees' comments but also raises considerable concerns.

As mentioned above, I have also sought further advice on the study, and our advisor reached a conclusion that "This paper ... is solid and the technologies used is up to date. I would consider its publication following last round of reviews." Therefore, I am pleased to inform you that we will be able to accept your manuscript pending the following final amendments:

1) Please address all the points raised by the referee #2 in writing as limitations of the study.
2) In the main manuscript file, please do the following:
- Correct/answer the track changes suggested by our data editors by working from the attached document.
- Make sure that all special characters display well.
- Add author contributions for Siti Naqiah Amrun and Bei Wang.
- Place "Data availability" at the end of M&M section.
3) Synopsis: Please check your synopsis text and image before submission and submit their final versions with your revised manuscript. Please be aware that in the proof stage minor corrections only are allowed (e.g., typos).
4) For more information: There is space at the end of each article to list relevant web links for further consultation by our readers. Could you identify some relevant ones and provide such information as well? Some examples are patient associations, relevant databases, OMIM/proteins/genes links, author's websites, etc...
5) Press release: Please inform us as soon as possible and latest at the time of submission of the revised manuscript if you plan a press release for your article so that our publisher could coordinate publication accordingly.
6) Please be aware that we use a unique publishing workflow for COVID-19 papers: a non-typeset PDF of the accepted manuscript is published as "Just Accepted" on our website. With respect to a possible press release, we have the option to not post the "Just Accepted" version if you prefer to wait with the press release for the typeset version. Please let us know whether you agree to publication of a "Just accepted" version or you prefer to wait for the typeset version.
7) As part of the EMBO Publications transparent editorial process initiative (see our Editorial at http://embomolmed.embopress.org/content/2/9/329), EMBO Molecular Medicine will publish online a Review Process File (RPF) to accompany accepted manuscripts. This file will be published in conjunction with your paper and will include the anonymous referee reports, your point-by-point response and all pertinent correspondence relating to the manuscript. Let us know whether you agree with the publication of the RPF and as here, if you want to remove or not any figures from it prior to publication. Please note that the Authors checklist will be published at the end of the RPF.
8) Please provide a point-by-point letter INCLUDING my comments as well as the reviewer's reports and your detailed responses (as Word file).

I look forward to reading a new revised version of your manuscript as soon as possible.

Yours sincerely,

Zeljko Durdevic

Zeljko Durdevic
Editor
EMBO Molecular Medicine

***** Reviewer's comments *****

Referee #2 (Remarks for Author):

The authors have provided a revised manuscript that addresses some of the concerns of the original reviewers. In particular, the changes made to the T cell analysis in light of the issues with the original gating are appreciated, and serve to provide increased confidence in the T cell data.

With regards to the concerns raised about the B cell analysis, I am unconvinced by aspects of the revised manuscript. The authors do clearly show that the elevated plasmablast response among breakthrough cases supports the presence of active infection, and implies that reactivation of the memory B cell response has already occurred at the timepoints sampled. Under those circumstances, it is therefore confusing to conclude that differences in RBD-specific MBC reflect the immunological state prior to infection, and contribute to susceptibility to breakthrough infection. It seems equally plausible that MBC have been recruited out of the circulation due to the presence of antigen in tissues or lymph nodes.

I would note in particular that the correlation between plasmablast frequency and RBD-specific MBC frequency in EV2 is quite unconvincing - it is described in the text on line 171 as a "trend toward positive correlation", but in a sample size of n=7 with one strongly positive outlying data point, this is an overstatement. Given the number of breakthrough cases available, this analysis seems quite underpowered.

Revised Submission Referee #2

Referee #2 (Remarks for Author):

The authors have provided a revised manuscript that addresses some of the concerns of the original reviewers. In particular, the changes made to the T cell analysis in light of the issues with the original gating are appreciated, and serve to provide increased confidence in the T cell data.

With regards to the concerns raised about the B cell analysis, I am unconvinced by aspects of the revised manuscript. The authors do clearly show that the elevated plasmablast response among breakthrough cases supports the presence of active infection, and implies that reactivation of the memory B cell response has already occurred at the timepoints sampled. Under those circumstances, it is therefore confusing to conclude that differences in RBD-specific MBC reflect the immunological state prior to infection, and contribute to susceptibility to breakthrough infection. It seems equally plausible that MBC have been recruited out of the circulation due to the presence of antigen in tissues or lymph nodes.

I would note in particular that the correlation between plasmablast frequency and RBD-specific MBC frequency in EV2 is quite unconvincing - it is described in the text on line 171 as a "trend toward positive correlation", but in a sample size of n=7 with one strongly positive outlying data point, this is an overstatement. Given the number of breakthrough cases available, this analysis seems quite underpowered.

Authors' Responses to Referee #2 (resubmission)

- *The authors have provided a revised manuscript that addresses some of the concerns of the original reviewers. In particular, the changes made to the T cell analysis in light of the issues with the original gating are appreciated, and serve to provide increased confidence in the T cell data.*

Response: We thank Referee #2 for the favourable evaluation of the revised manuscript, especially for the revised T cell analysis.

- *With regards to the concerns raised about the B cell analysis, I am unconvinced by aspects of the revised manuscript. The authors do clearly show that the elevated plasmablast response among breakthrough cases supports the presence of active infection, and implies that reactivation of the memory B cell response has already occurred at the timepoints sampled. Under those circumstances, it is therefore confusing to conclude that differences in RBD-specific MBC reflect the immunological state prior to infection, and contribute to susceptibility to breakthrough infection. It seems equally plausible that MBC have been recruited out of the circulation due to the presence of antigen in tissues or lymph nodes.*

Response: We agree that the current study design does not allow us to completely rule out the possibility that MBC have been recruited out of the circulation – this would require either a prospective study design or extensive tissue sampling, both of which are out of the scope of the current study. We have previously addressed this limitation in the discussion (lines 262-266). We have now revised the manuscript to further emphasize this limitation and the possible alternative interpretation by adding the following in line 266-268: "In particular,

reduced memory B cell levels in vaccine breakthrough cases may have been due to recruitment of memory B cells out of circulation after activation by infection."

- *I would note in particular that the correlation between plasmablast frequency and RBD-specific MBC frequency in EV2 is quite unconvincing - it is described in the text on line 171 as a "trend toward positive correlation", but in a sample size of n=7 with one strongly positive outlying data point, this is an overstatement. Given the number of breakthrough cases available, this analysis seems quite underpowered.*

Response: We were unfortunately unable to get paired plasmablast-memory B cell data for a number of the patients due to sample limitations as well as some technical difficulties. We agree that the current graph is strongly influenced by a strong positive data point; however, even if this point is removed, the remaining data still do not show a negative correlation between plasmablasts and memory B cells (see Figure EV3 with strong positive point removed; right). The lack of negative correlation is one piece of evidence against the alternate theory that MBCs are being lost via differentiation into plasmablasts. In light of this, we have now deleted the sentences regarding the trend toward positive correlation and its interpretation, and we have chosen to focus on the lack of inverse correlation instead (lines 166-169).

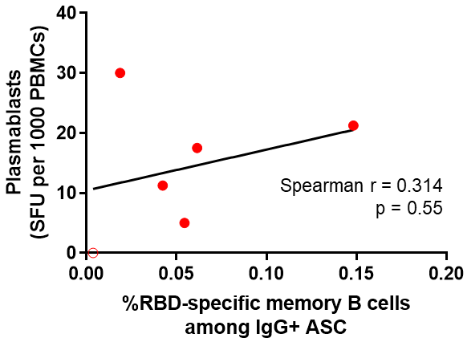

Figure EV3 with strong positive point removed.

We are pleased to inform you that your manuscript is accepted for publication and is now being sent to our publisher to be included in the next available issue of EMBO Molecular Medicine.

EMBO PRESS

YOU MUST COMPLETE ALL CELLS WITH A PINK BACKGROUND ➔

PLEASE NOTE THAT THIS CHECKLIST WILL BE PUBLISHED ALONGSIDE YOUR PAPER

Corresponding Author Name: Lisa F. P. Ng	
Journal Submitted to: EMBO Molecular Medicine	
Manuscript Number: EMM-2021-15227	

Reporting Checklist For Life Sciences Articles (Rev. June 2017)

This checklist is used to ensure good reporting standards and to improve the reproducibility of published results. These guidelines are consistent with the Principles and Guidelines for Reporting Preclinical Research issued by the NIH in 2014. Please follow the journal's authorship guidelines in preparing your manuscript.

A- Figures

1. Data

The data shown in figures should satisfy the following conditions:

- ➔ the data were obtained and processed according to the field's best practice and are presented to reflect the results of the experiments in an accurate and unbiased manner.
- ➔ figure panels include only data points, measurements or observations that can be compared to each other in a scientifically meaningful way.
- ➔ graphs include clearly labeled error bars for independent experiments and sample sizes. Unless justified, error bars should not be shown for technical replicates.
- ➔ if n< 5, the individual data points from each experiment should be plotted and any statistical test employed should be justified
- ➔ Source Data should be included to report the data underlying graphs. Please follow the guidelines set out in the author ship guidelines on Data Presentation.

2. Captions

Each figure caption should contain the following information, for each panel where they are relevant:

- ➔ a specification of the experimental system investigated (eg cell line, species name).
- ➔ the assay(s) and method(s) used to carry out the reported observations and measurements
- ➔ an explicit mention of the biological and chemical entity(ies) that are being measured.
- ➔ an explicit mention of the biological and chemical entity(ies) that are altered/varied/perturbed in a controlled manner.

- ➔ the exact sample size (n) for each experimental group/condition, given as a number, not a range;
- ➔ a description of the sample collection allowing the reader to understand whether the samples represent technical or biological replicates (including how many animals, litters, cultures, etc.).
- ➔ a statement of how many times the experiment shown was independently replicated in the laboratory.
- ➔ definitions of statistical methods and measures:
  - common tests, such as t-test (please specify whether paired vs. unpaired), simple χ2 tests, Wilcoxon and Mann-Whitney tests, can be unambiguously identified by name only, but more complex techniques should be described in the methods section;
  - are tests one-sided or two-sided?
  - are there adjustments for multiple comparisons?
  - exact statistical test results, e.g., P values = x but not P values < x;
  - definition of 'center values' as median or average;
  - definition of error bars as s.d. or s.e.m.

Any descriptions too long for the figure legend should be included in the methods section and/or with the source data.

In the pink boxes below, please ensure that the answers to the following questions are reported in the manuscript itself. Every question should be answered. If the question is not relevant to your research, please write NA (non applicable). We encourage you to include a specific subsection in the methods section for statistics, reagents, animal models and human subjects.

USEFUL LINKS FOR COMPLETING THIS FORM

http://www.antibodypedia.com	Antibodypedia
http://1degreebio.org	1DegreeBio
http://www.equator-network.org/reporting-guidelines/improving-bioscience-research-repor	ARRIVE Guidelines
http://grants.nih.gov/grants/olaw/olaw.htm	NIH Guidelines in animal use
http://www.mrc.ac.uk/Ourresearch/Ethicsresearchguidance/Useofanimals/index.htm	MRC Guidelines on animal use
http://ClinicalTrials.gov	Clinical Trial registration
http://www.consort-statement.org	CONSORT Flow Diagram
http://www.consort-statement.org/checklists/view/32-consort/66-title	CONSORT Check List
http://www.equator-network.org/reporting-guidelines/reporting-recommendations-for-tumo	REMARK Reporting Guidelines (marker prognostic studies)
http://datadryad.org	Dryad
http://figshare.com	Figshare
http://www.ncbi.nlm.nih.gov/gap	dbGAP
http://www.ebi.ac.uk/ega	EGA
http://biomodels.net/	Biomodels Database
http://biomodels.net/miriam/	MIRIAM Guidelines
http://jjj.biochem.sun.ac.za	JWS Online
https://osp.od.nih.gov/biosafety-biosecurity-and-emerging-biotechnology/	Biosecurity Documents from NIH
http://www.selectagents.gov/	List of Select Agents

B- Statistics and general methods

Please fill out these boxes ➔ (Do not worry if you cannot see all your text once you press return)

1.a. How was the sample size chosen to ensure adequate power to detect a pre-specified effect size?	No statistical methods were used to calculate sample size. Sample size for vaccine breakthrough patients and close contacts was determined based on the number of patients that were enrolled and consented with the current study between Apr-Sep 2021.
1.b. For animal studies, include a statement about sample size estimate even if no statistical methods were used.	NA - no animal studies
2. Describe inclusion/exclusion criteria if samples or animals were excluded from the analysis. Were the criteria pre-established?	Some samples were excluded from some assays due to lack of sample availability, or assay failure. Criteria for determining assay failure were pre-established.
3. Were any steps taken to minimize the effects of subjective bias when allocating animals/samples to treatment (e.g. randomization procedure)? If yes, please describe.	No animal studies. For patient samples, no randomization was required, since all available samples from eligible enrolled participants were used.
For animal studies, include a statement about randomization even if no randomization was used.	NA - no animal studies
4.a. Were any steps taken to minimize the effects of subjective bias during group allocation or/and when assessing results (e.g. blinding of the investigator)? If yes please describe.	Investigators were not blinded to group allocation during data collection and analysis, because samples from vaccine breakthrough patients had to be handled with additional safety precautions.
4.b. For animal studies, include a statement about blinding even if no blinding was done	NA - no animal studies
5. For every figure, are statistical tests justified as appropriate?	Yes.
Do the data meet the assumptions of the tests (e.g., normal distribution)? Describe any methods used to assess it.	Non-parametric tests were used in all cases.
Is there an estimate of variation within each group of data?	Yes - median +- interquartile range are given for all graphs.
Is the variance similar between the groups that are being statistically compared?	NA - non-parametric tests used.

C- Reagents

6. To show that antibodies were profiled for use in the system under study (assay and species), provide a citation, catalog number and/or clone number, supplementary information or reference to an antibody validation profile. e.g., Antibodypedia (see link list at top right), 1DegreeBio (see link list at top right).	Marker Clone<br>Cat. No. CD66BG10F5<br>562940CD45RAH10062-0458-42<br>CD27O323302822<br>NKG2C134591748168<br>CD8SK1564116<br>CD19HIB19740394<br>VD2B6331412<br>CD107AH4A3563869<br>NKP469-e2564536<br>CD3SK7344852<br>CD1697-239346020<br>HLADRL243307626<br>CD4SK3344658<br>CD14M0P9557831<br>VD1REA173130-120-578<br>CXCR5J252D4356928<br>CD154TRAP-1555701<br>CD56NCAM16.235-0567-42<br>CD163G8MHCD1624<br>CD45H130612891<br>Granzyme BGB11563388<br>IFN-y4S.B3502534<br>IL-2MQ1-17H12564166<br>TNF-aMAB11566359<br>IL-6MQ2-13A511-7069-82<br>IL-17aN49-653560490<br>IL-10JES-9D7501404<br>IL-48D4-8560672
7. Identify the source of cell lines and report if they were recently authenticated (e.g., by STR profiling) and tested for mycoplasma contamination.	HEK-293T stably transduced with SARS-CoV-2 Spike antigen were made by Y.S. Goh; original HEK-293T cells from ATCC.<br>CHO cells stably transduced with ACE2 were a gift from Professor Yee-Joo Tan, Department of Microbiology, NUS & IMCB, A*STAR, Singapore.<br>The cell lines tested negative for contamination with mycoplasma.

* for all hyperlinks, please see the table at the top right of the document

D- Animal Models

8. Report species, strain, gender, age of animals and genetic modification status where applicable. Please detail housing and husbandry conditions and the source of animals.	NA - no animal studies
9. For experiments involving live vertebrates, include a statement of compliance with ethical regulations and identify the committee(s) approving the experiments.	NA - no animal studies
10. We recommend consulting the ARRIVE guidelines (see link list at top right) (PLoS Biol. 8(6), e1000412, 2010) to ensure that other relevant aspects of animal studies are adequately reported. See author guidelines, under 'Reporting Guidelines'. See also: NIH (see link list at top right) and MRC (see link list at top right) recommendations. Please confirm compliance.	NA - no animal studies

E- Human Subjects

11. Identify the committee(s) approving the study protocol.	The study design and protocols for the COVID-19 PROTECT study group were evaluated by National Healthcare Group (NHG) Domain Specific Review Board (DSRB) and approved under study number 2012/00917. Collection of healthy donor samples was approved by SingHealth Centralized Institutional Review Board (CIRB) under study number 2017/2806 and NUS IRB 04-140.
12. Include a statement confirming that informed consent was obtained from all subjects and that the experiments conformed to the principles set out in the WMA Declaration of Helsinki and the Department of Health and Human Services Belmont Report.	Written informed consent was obtained from participants in accordance with the Declaration of Helsinki for Human Research. The experiments conformed to the principles set out in the Department of Health and Human Services Belmont Report.
13. For publication of patient photos, include a statement confirming that consent to publish was obtained.	NA - no patient photos used
14. Report any restrictions on the availability (and/or on the use) of human data or samples.	Relevant data are given in the Source Data files. Other data are available on reasonable request from the corresponding author, LFPN. Patient data are not publicly available due to containing information that could compromise the privacy of research participants.
15. Report the clinical trial registration number (at ClinicalTrials.gov or equivalent), where applicable.	NA - not a clinical trial
16. For phase II and III randomized controlled trials, please refer to the CONSORT flow diagram (see link list at top right) and submit the CONSORT checklist (see link list at top right) with your submission. See author guidelines, under 'Reporting Guidelines'. Please confirm you have submitted this list.	NA
17. For tumor marker prognostic studies, we recommend that you follow the REMARK reporting guidelines (see link list at top right). See author guidelines, under 'Reporting Guidelines'. Please confirm you have followed these guidelines.	NA

F- Data Accessibility

18: Provide a "Data Availability" section at the end of the Materials & Methods, listing the accession codes for data generated in this study and deposited in a public database (e.g. RNA-Seq data: Gene Expression Omnibus GSE39462, Proteomics data: PRIDE PXD000208 etc.) Please refer to our author guidelines for 'Data Deposition'.<br><br>Data deposition in a public repository is mandatory for:<br>a. Protein, DNA and RNA sequences<br>b. Macromolecular structures<br>c. Crystallographic data for small molecules<br>d. Functional genomics data<br>e. Proteomics and molecular interactions	NA - no large datasets generated.<br>Relevant data are given in the Source Data files. Other data are available on reasonable request from the corresponding author, LFPN. Patient data are not publicly available due to containing information that could compromise the privacy of research participants.
19. Deposition is strongly recommended for any datasets that are central and integral to the study; please consider the journal's data policy. If no structured public repository exists for a given data type, we encourage the provision of datasets in the manuscript as a Supplementary Document (see author guidelines under 'Expanded View' or in unstructured repositories such as Dryad (see link list at top right) or Figshare (see link list at top right).	NA - no large datasets generated.
20. Access to human clinical and genomic datasets should be provided with as few restrictions as possible while respecting ethical obligations to the patients and relevant medical and legal issues. If practically possible and compatible with the individual consent agreement used in the study, such data should be deposited in one of the major public access-controlled repositories such as dbGAP (see link list at top right) or EGA (see link list at top right).	NA - no genomic datasets generated
21. Computational models that are central and integral to a study should be shared without restrictions and provided in a machine-readable form. The relevant accession numbers or links should be provided. When possible, standardized format (SBML, CellML) should be used instead of scripts (e.g. MATLAB). Authors are strongly encouraged to follow the MIRIAM guidelines (see link list at top right) and deposit their model in a public database such as Biomodels (see link list at top right) or JWS Online (see link list at top right). If computer source code is provided with the paper, it should be deposited in a public repository or included in supplementary information.	NA - no computational models developed

G- Dual use research of concern

22. Could your study fall under dual use research restrictions? Please check biosecurity documents (see link list at top right) and list of select agents and toxins (APHIS/CDC) (see link list at top right). According to our biosecurity guidelines, provide a statement only if it could.	No
